# Eye’ll Help You Out! How the Gaze Cue Reduces the Cognitive Load Required for Reference Processing

**DOI:** 10.1111/cogs.12682

**Published:** 2018-10-07

**Authors:** Mirjana Sekicki, Maria Staudte

**Affiliations:** ^1^ Department of Language Science and Technology Saarland University

**Keywords:** Situated sentence comprehension, Visual world paradigm, Referential gaze, Anticipatory eye movements, Verbal restrictions, Surprisal, Cognitive load, The Index of Cognitive Activity

## Abstract

Referential gaze has been shown to benefit language processing in situated communication in terms of shifting visual attention and leading to shorter reaction times on subsequent tasks. The present study simultaneously assessed both visual attention and, importantly, the immediate cognitive load induced at different stages of sentence processing. We aimed to examine the dynamics of combining visual and linguistic information in creating anticipation for a specific object and the effect this has on language processing. We report evidence from three visual‐world eye‐tracking experiments, showing that referential gaze leads to a shift in visual attention toward the cued object, which consequently lowers the effort required for processing the linguistic reference. Importantly, perceiving and following the gaze cue did not prove costly in terms of cognitive effort, unless the cued object did not fit the verb selectional preferences.

## 1 Introduction

If the comprehension of a particular linguistic item relies on its immediate (linguistic) context, in face‐to‐face communication that context is further enriched by the visual information. Here, the likely referents for a linguistic expression may be among the co‐present visual objects. The concrete set of potential referents can then be further reduced by using visual cues such as gestures or gaze, in order to avoid ambiguities and facilitate language processing.

Previous research has examined what is being activated and predicted during visually situated language processing (e.g., Altmann & Kamide, 1999), whether gaze creates a shift in viewers’ attention (Friesen & Kingstone, 1998), its effect on listeners’ language comprehension (Hanna & Brennan, 2007), and how beneficial such a cue is for processing subsequent linguistic material (e.g., Staudte & Crocker, 2011). Referential speaker gaze cue, as a visual pointer, directs listeners’ visual attention to the cued object, which was consequently shown to be facilitatory, as measured by listeners’ performance on post‐trial tasks. Previous research relied on tracking eye movements, as an online measure of visual attention, and post‐trial task performance, as an offline indication of processing difficulty. In the present paper, we extend these findings by measuring processing effort online. We employ a pupillary measure, the Index of Cognitive Activity (ICA), that allows for (a) inspection of free anticipatory eye movements, and (b) an online record of cognitive load at various points of interest throughout the sentence, where relevant linguistic and visual stimuli are introduced. We assessed how anticipatory eye movements connect to immediate cognitive effort, that is, whether anticipating objects (due to gaze‐following) facilitates the processing of their reference. Unlike previous research that has examined the benefit of gaze offline, by measuring post‐trial task performance, we approach the question more directly by reporting cognitive load required for processing the linguistic reference. Moreover, we are interested in examining whether perceiving the gaze cue itself, that is, processing the cued object, actually induces cognitive effort, on a par with the cost of linguistic processing.^1^

First, we manipulated the existence of the referential speaker gaze cue and assessed its effect on referent noun processing (Experiment 1). Also, we measured cognitive load on the gaze cue itself, expecting a distribution of load between the cue and the reference, such that the reduction of cognitive load on the referent noun is preceded by its increase on the gaze cue. Thus, we examined not only the effect that gaze‐following may have on processing the subsequent reference, but also the cognitive load required for the shift in visual attention, where both linguistic and visual information are being considered.

Second, in order to better understand the mechanisms behind gaze perception and its integration with language processing, we manipulated the context fit of the gaze cue (Experiment 2) and its congruency with the following referent noun (Experiment 3). We expected that the gaze cue would induce significantly higher cognitive load when it did not match the previous linguistic context, but that it would nevertheless still facilitate processing of the referent noun. Finally, the incongruent gaze cue was expected to be costly for the processing of the linguistic reference.

### 1.1 Prediction in language processing

There have been attempts to explain the intriguing ease with which people use language in terms of prediction, or rather, the probability of an element to follow the current state, first and foremost, based on the immediate linguistic context. Each element constrains the choice of the subsequent one, which is the information used to anticipate what is likely to follow. In the same vein, in situated communication, the immediate visual context provides additional information that increases the probability of a concrete, visually present entity to be referred to by language. In addition, visual pointers are commonly used to further increase the probability of a particular entity, and hence, resolve ambiguities and help avoid misunderstandings.

Inspired by Shannon (1948)'s notion of surprisal (see Hale, 2001; Levy, 2008), studies have connected sentences with varyingly surprising continuations with different measures of processing difficulty. Reading patterns (e.g., Rayner & Well, 1996), reading times (e.g., Demberg & Keller, 2008), and the N400 ERP component (e.g., Federmeier & Kutas, 1999; Frank, Otten, Galli, & Vigliocco, 2013) have shown that lower probability of a linguistic item leads to higher processing cost. For more on prediction in language processing, please see a recent discussion by Kuperberg and Jaeger (2016).

The visual world paradigm (VWP) has been used extensively to employ anticipatory eye movements to visually present entities that give insight into what is being activated during listening. Kamide, Altmann, and Haywood (2003) showed that verb information is utilized to anticipate upcoming arguments, and vice versa (Japanese study). Many specific questions have been examined, for instance, if it is particular words that people predict and thus their phonological features (Allopenna, Magnuson, & Tanenhaus, 1998), or whether specific visual features (Dahan & Tanenhaus, 2005) such as shape of objects are being activated (Huettig & Altmann, 2007; Rommers, Meyer, Praamstra, & Huettig, 2013), or object affordances (Altmann & Kamide, 2007), or even objects that are semantically related, but non‐associated with the target (Huettig & Altmann, 2004).

It has been shown that both available linguistic and visual information are combined and utilized to predict the upcoming linguistic content. In addition, the eye movement analysis that the paradigm supports showed active updating of the mental representation, based on both linguistic and visual information (see Altmann & Mirkovic, 2009; Huettig, Rommers, & Meyer, 2011).

An additional notion relevant for our present work is that of Uniform Information Density (UID):

Within the bounds defined by grammar, speakers prefer utterances that distribute information uniformly across the signal (information density). Where speakers have a choice between several variants to encode their message, they prefer the variant with more uniform information density (ceteris paribus). (Jaeger, 2010, p. 3)

We hypothesized that both linguistic and visual context contribute to the distribution of information, and that visual cues allow for a more uniform distribution throughout a sentence.

**Figure 1:**
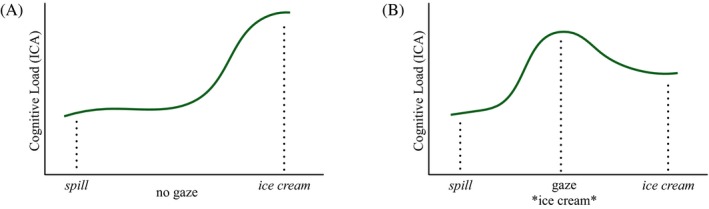
Expected distribution of cognitive load among the visual and linguistic cues (assuming that gaze is typically informative). (a) The distribution of cognitive load without the gaze cue. (b) Cognitive load distribution with the gaze cuing the target object.

Present work assumes a direct link between cognitive load, as measured by the ICA (see Section 1.3 for a description of the measure), and the surprisal of the referent noun. Our predictions are in line with the information‐theoretic notions of probability and surprisal, in that we expect that the higher the probability of an item, given its previous linguistic and visual context, the lower the surprisal on the actual referent will be. Thus, we expect lower cognitive load on the referent noun when it is in accordance with the predictions previously made based on linguistic and visual information. Moreover, inspired by the UID hypothesis, we expect that the information provided through the gaze cue affects the immediate cognitive load it induces. An informative gaze cue that leads to the reduction of load on the referent noun is thus expected to induce higher cognitive load itself. In this way, the processing of the same information content would be distributed between the visual cue and the noun. This idea is illustrated in Fig. 1. We assume that the noun *ice cream*, in a sentence like *The man spills the ice cream*, would induce relatively high cognitive load, since in order to be a logical argument of the verb *spill*, ice cream would have to be melted, which is not its typical state. However, if the speaker were to look at the ice cream, an object present in the shared visual context, prior to uttering the reference, both the gaze cue and the referent noun would essentially introduce the same piece of information. Thus, it would be reasonable to expect, first, a reduction of effort on the reference, and second, that it is preceded by an increase of the load at the gaze cue. In sum, we expect a re‐distribution of the effort between the visual and linguistic cues.

### 1.2 The gaze cue

The information gathered from interlocutors’ gaze has proven to be an inseparable part of situated communication. Interlocutors spend a lot of time looking at each other (Argyle & Cook, 1976), the listener being the one who spends more time looking at the speaker (Kendon, 1967), while mutual gaze is utilized to coordinate turn‐taking (Duncan, 1972; Kendon, 1967). Situated communication commonly includes visual cues, such as pointing, that are used to disambiguate and ground referring expressions (Bangerter, 2004). In their seminal work, Hanna and Brennan (2007) show that a speaker's eye gaze can similarly be used to resolve temporary ambiguity, concluding that it is a conversationally based source of information used to quickly constrain reference resolution.

Listeners inspect objects they anticipate will be mentioned next (Altmann & Kamide, 1999), and additionally fixate the mentioned object 200–300 ms after the onset of the corresponding reference (e.g., Allopenna et al., 1998; Tanenhaus, Spivey‐Knowlton, Eberhard, & Sedivy, 1995). Moreover, speakers also attend to the relevant object 800–1,000 ms before referring to it (e.g., Griffin & Bock, 2000; Meyer, Sleiderink, & Levelt, 1998). The speaker's gaze on an object present in the shared visual context thus provides a cue to her focus of attention (Emery, 2000; Flom, Lee, & Muir, 2007). This cue is then actively used to facilitate linguistic processing by helping to ground and disambiguate referring expressions (e.g., Hanna & Brennan, 2007). This also holds in communication with a robot speaker (Staudte & Crocker, 2011) or a virtual agent (Staudte, Crocker, Heloir, & Kipp, 2014), showing that people establish basic joint attention with robots as well, though this is dependent on belief in their competence (Staudte & Crocker, 2011).

The facilitatory effect of the speaker referential gaze cue has been established in previous literature (e.g., Knoeferle & Kreysa, 2012; Macdonald & Tatler, 2013, 2014; Staudte & Crocker, 2011; Staudte et al., 2014). Importantly, though, the gaze effect on the processing cost of the linguistic referent has been measured only offline, via task performance measures such as accuracy and reaction times. Only recently, Jachmann, Drenhaus, Staudte, and Crocker (2017) examined the effect of speaker referential gaze cue on the processing of the linguistic reference, by considering event‐related potentials. Their results suggest that gaze led to anticipation of the upcoming noun, which resulted in integration difficulty when the cue was incongruent with the sentence. Moreover, they found evidence that gaze inspires anticipation of specific word forms.

The present paper presents a group of studies that pioneer in measuring directly online both listeners’ visual attention, as well as cognitive effort induced (a) by perceiving the speaker's referential gaze cue, and (b) by processing the linguistic reference. We hypothesized that the information gathered from both visual and linguistic cues is incrementally combined to create anticipation for upcoming linguistic material. Since the same piece of information is conveyed by the gaze cue and the referent noun, gaze being the first to appear, we expected it to induce more cognitive load, which would consequently reduce the load on the linguistic reference, and thus, result in a distribution of cognitive effort between the two modalities. In addition, we were interested in examining the facilitatory effect of the gaze cue in atypical contexts created through either a mismatch with verb selectional preferences, or incongruency with the subsequent linguistic reference.

The present experiments made use of controlled visual items with the gaze cue that was represented by a simple line drawing. Since the gaze cue was presented simultaneously with the linguistic material, in a manner in which speakers typically use their gaze cue, we believe that this inspires the perception of the cue as being related to the speaker. Post‐experimental debriefing showed that participants indeed anthropomorphized the presented drawing, perceiving it as a face. This is discussed further in Section 5.

### 1.3 Pupillometry and language processing

Pupil size is proven to be a reliable measure of cognitive effort induced by language processing. Moreover, it has been shown to reflect the effect of the visual context on processing load during comprehension. For instance, examining processing effort for spoken garden path sentences, Engelhardt, Ferreira, and Patsenko (2010) showed that when visual context was consistent with the correct sentence interpretation, sentence prosody had little effect on processing load. Also, they found evidence that increased effort did not result in more incorrect sentence interpretations, concluding that pupil size is a more sensitive measure of language processing than task performance. Another study in the VWP showed that when the visual context disambiguated the initial NP as the object of a sentence (OVS word order), listeners’ pupils became significantly more dilated than when the visual context supported the anticipation of SVO word order (Scheepers & Crocker, 2004). For a full account of the existing literature employing pupillometry in linguistic research, please see a recent review by Schmidtke (2017).

As presented by Beatty and Lucero‐Wagoner (2000), pupil size is determined by the activity of two opposing muscle groups within the iris. The contraction of the circular muscles constricts the pupil, while the activity of radial muscles makes the pupil dilate. Pupil dilation can happen as a consequence of changes in light, where the pupillary light reflex regulates the amount of light entering the eye. Moreover, dilation also occurs due to sensory, mental, and emotional events. Different causes of dilation employ different activation and inhibition processes, the dilation due to cognitive load being shorter and more abrupt than that caused by the light reflex.

Task‐evoked pupillary responses (TEPRs), such as mean pupil dilation, peak dilation, and latency to peak, are established as a reliable pupillary measure of cognitive load in psychological research. They are obtained by a time‐locked averaging of the pupillary record with respect to critical events in a task (Beatty, 1982). Importantly, though, the light reflex causes pupilar dilation larger than that induced by mental events, which is a potential confound that this technique cannot account for. Thus, effort has to be made to avoid masking smaller TEPRs by such optic reflexes (Beatty & Lucero‐Wagoner, 2000), by keeping constant not only the luminance level in the experimental room, but also the luminance level of visual stimuli. As noted by Demberg and Sayeed (2016), this is not an easy task, since the pupil exhibits irregular oscillation under the influence of constant light. Moreover, even fixating darker or lighter objects in a scene can affect overall pupil size (Demberg & Sayeed, 2016). Interestingly, there is evidence that the pupillary light reflex can be artificially induced in the condition of constant luminance by showing pictures that suggest brightness, for instance by contrasting images of the sun and the moon (Binda, Pereverzeva, & Murray, 2013; Laeng & Endestad, 2012; Naber & Nakayama, 2013). Another potential confound that has to be accounted for is that the pupil size can vary due to participants’ gaze position (see Hayes & Petrov, 2016; Scheepers & Crocker, 2004). Finally, it is important to note that pupil response is relatively slow to return to baseline (Schmidtke, 2017).

In the past few years, another pupillary measurement has attracted attention by proposing to solve these issues. The ICA performs a wavelet analysis on the pupil dilation record by disentangling the change in pupil size due to cognitive load from that induced by the light reflex (Marshall, 2000). The index reflects unusual increases in the pupil signal occurring due to effortful cognitive activity, and it is computed as the number of times per second that an abrupt discontinuity in the pupil signal is detected (Marshall, 2007). These events of small abrupt changes in pupil size are referred to as the *ICA events*. Low values per second indicate lower cognitive effort, while high values, conversely, reflect more cognitive activity. Importantly, the measurement maintains both time and frequency information, so that the exact time at which an ICA event was observed is detectable.

Since its appearance, the ICA has been tested in a variety of different cognitive tasks (e.g., Marshall, 2002, 2007; Schwalm, Keinath, & Zimmer, 2008). Recently, it has also been examined with cognitive load induced by linguistic processing (Demberg & Sayeed, 2016; Tourtouri, Delogu, & Crocker, 2017). Demberg and Sayeed (2016) employed the ICA in a series of seven experiments, with different kinds of linguistic stimuli and different modes of stimulus presentation. The ICA proved to reflect linguistically induced cognitive load for both reading and auditory presentation of linguistic material. In addition, experiments in the driving simulator and the VWP revealed that the measurement is robust with respect to eye movements and lumination changes. Demberg and Sayeed (2016) conclude that the ICA is applicable as a measure of processing effort that does not have to account for artefacts due to eye movements or changes in screen luminocity, deeming it a promising measure for simultaneous assessment of visual attention and processing difficulty. Recently, Tourtouri et al. (2017) obtained reliable results by doing just that, employing the ICA measurement in the VWP in combination with eye movement analysis.

Having considered all of the above, and since the aim of the present work required the simultaneous assessment of both visual attention (i.e., free eye movements) and cognitive load, we have decided to design our experiments in the VWP to employ the eye‐tracking technology together with the discussed pupillary measure of cognitive load. Hence, in the following, we report and interpret anticipatory eye movements, and the analysis of the ICA. For a fine‐grained analysis of cognitive load we use the raw ICA workload, that is, the output including information of the exact timing of each ICA event.

### 1.4 Experiments

Three experiments conducted in the VWP measured eye movements and cognitive load reflected in pupil size. We paired simple SVAdvO sentences in the German language with visual displays of four objects and a stylized gaze cue. The gaze cue was presented together with the adverb, that is, after the verb had been introduced and before the introduction of the referent noun.

The eye movement analysis revealed patterns of anticipation and visual attention, while employing the ICA allowed us to measure immediate cognitive load induced both by the perception of the gaze cue and by processing the referent noun, that is, the cognitive load induced by creating and (dis)confirming one's predictions.

**Figure 2:**
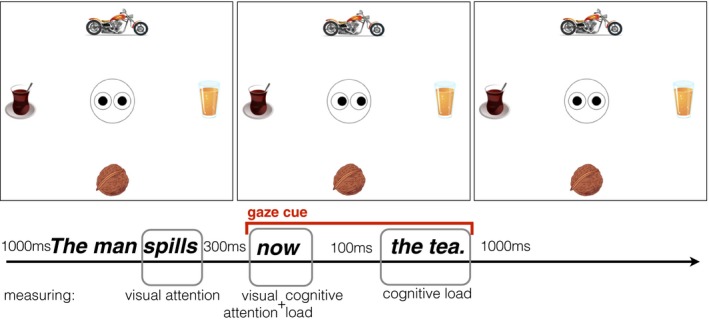
Example timeline illustrating an incongruent gaze cue (Exp. 2). Note that the sentence shown is a literal translation of the German sentence used for the experiment and preserves the exact word order.

As illustrated by Fig. 2, we examined visual attention at the verb—the point where verb selectional preferences allow for discarding some of the depicted objects and activating those with the highest probability to appear in the continuation of the sentence. Second, we considered visual attention at the point of introducing the gaze cue—examining whether it is being followed, causing a shift in visual attention. In addition, interested in whether perceiving an object after such an attention shift is costly, we measured immediate cognitive load at the gaze cue. Finally, we considered cognitive load induced by the referent noun.

The present studies address the following questions:

1 Does the gaze cue influence the predictability of a linguistic reference?
2 If so, how does this influence the cognitive load (surprisal) on the reference?
a) How does a gaze cue affect cognitive load on the following reference when the cued object does not fit the previous linguistic context?
b) Would a gaze cue, fitting with the previous linguistic context but incongruent with the following reference, affect the cognitive load on the (also fitting) referent noun?
3 Is the gaze cue costly in itself?
a) Is the gaze cue to a mismatching object costly?
4 Is there a distribution of cognitive load between the gaze cue and the referent noun, since they both select the same target referent?

Experiment 1 aims to answer the main four questions, while the other two experiments address the more specific subquestions, by first replicating the findings of Experiment 1 in a slightly different setting, and second, giving more detailed answers to the main research questions.

In sum, we looked at anticipatory eye movements and measured direct online effort at various points of interest throughout the sentence. Experiment 1 shows the facilitatory effect of the gaze cue on processing the subsequent referent noun. Experiment 2 challenges this finding and examines if facilitation of gaze holds even when the target referent does not fit the linguistic context. And finally, Experiment 3 examines the effects of gaze congruency, that is, if gaze cuing an object that is later not mentioned induces cost on the referent noun, even though the reference fits the context.

In the context of the present paper, we use the word *gaze* to refer to the referential gaze cue presented by the stylized faze (speaker). In contrast, *inspections* and *fixations* are used to refer to the listeners’ visual attention.

## 2 Experiment 1

This study aimed to examine whether employing the ICA measurement supports previous findings that the gaze cue is actively considered in language processing, by quantifying online how the existence of the gaze cue modifies the cognitive load induced by the linguistic reference. More important, we were interested in measuring the potential cost of gaze perception and the distribution of cognitive load between the gaze cue and the linguistic reference.

The gaze cue used in this study was always **fitting** (with the previous linguistic context) and **congruent** (cuing the object subsequently referred to by language). We manipulated the existence of the gaze cue in order to answer the four main research questions (listed on p. 8).

We expected gaze to be followed, and thus inform anticipation of the object that is likely to be mentioned next. Consequently, this was expected to lead to lower cognitive load when the anticipated object was finally referred to. In addition, we expected the perception of the gaze cue not to be costly as such, but that higher cognitive load would be measured on the gaze cue when it is not consistent with the expectation already created based on the previous linguistic context (verb). Finally, this was expected to lead to a reduction of cognitive load on the referent noun, since the effort required to process an unexpected reference would have been distributed between the gaze cue and the referent noun.

### 2.1 Method

The study made use of 2 × 2 × 2 mixed factorial design. The independent variable **Gaze** (no‐gaze vs. referent gaze) was a between‐subjects variable; that is, half of the participants were presented with the version of the experiment where all items included the gaze cue, while the other half saw the version with item trials never having the gaze cue. Fillers balanced the gaze conditions in the experiment as a whole to the ratio of 1:1, so that all participants saw gaze and no‐gaze trials. In addition, four linguistic conditions were created with two within‐subjects variables, **Constraint** (restrictive vs. non‐restrictive) and **Plausibility** (plausible vs. possible). Constraint was manipulated by verb restrictiveness (*spill* vs. *order*), and Plausibility by noun fit with the restrictive verb (*spill water* vs. *spill ice cream*). The language of the experiment was German.

#### 2.1.1 Participants

Sixty‐four Saarland University students took part in this study (45 women) and were monetarily reimbursed for their participation. Their ages ranged from 18 to 34 years (*M* = 24.16). Participants were all native speakers of the German language with normal or corrected‐to‐normal vision.

#### 2.1.2 Items

Each participant was presented with 20 items and 30 fillers, consisting of static visual scenes and auditorily presented linguistic stimulus. In addition, visual scenes included a face‐like object forming the gaze cue.

We used syntactically simple sentences with the SVAdvO structure. An item would include either a restrictive (*spill*) or a non‐restrictive (*order*) verb. Next, an adverb (*gleich*—literal translation: *now*) was included as a spill‐over region between the verb and the referent noun, where no relevant additional information is introduced. Rather, time is given for processing the verb and creating potential anticipations for the referent noun. The adverb was kept the same for all item sentences. In addition, this is the region where the gaze cue was introduced, making the point of the gaze cue subsequent to the constraining information introduced by the verb and prior to the resolution brought about with the referent noun. Finally, the object noun was introduced. Two linguistic conditions were created based on the noun's semantic fit in relation to the restrictive verb (*spill water* vs. *spill ice cream*).

In order to control for the frequency of different noun referents, we first chose a group of concrete inanimate nouns of roughly the same frequency derived from DeReWo word lists of the German Reference Corpus (DeReWo, 2012). We then created the stimuli sentences and subsequently conducted two pretests in order to check whether our manipulation was successful.

First, we conducted a plausibility rating, where stimuli were rated on a 7‐point Likert scale, where 1 was set to “highly plausible” and 7 to “not plausible at all.” Fourteen German native speakers completed the questionnaire (18–58 years of age). The dependent variable Rating Score was treated as a count variable; thus, we used generalized mixed effects models of Poisson type. Maximal converging random structure was included (Barr, Levy, Scheepers, & Tily, 2013). Also, independent variables were contrast coded before the analysis. The full model^2^ revealed a significant interaction between Constraint and Plausibility (β = −1.034, *SE* = 0.101, *z* = −10.271, *p* < .001), as well as a main effect of Plausibility (β = −0.654, *SE* = 0.058, *z* = 11.355, *p* < .001). Further comparisons^3^ showed a main effect of Plausibility in the subset of the restrictive verb (β = −1.093, *SE* = 0.127, *z* = 8.616, *p* < .001), suggesting that *spill water* (*M* = 1.043, *SD* = 0.346) was rated as more plausible than *spill ice cream* (*M* = 3.421, *SD* = 2.247). In addition, in the subset of the non‐restrictive verb we found no effect of Plausibility (*p* = .167), suggesting that *order water* (*M* = 1.679, *SD* = 1.583) was not rated differently from *order ice cream* (*M* = 1.964, *SD* = 1.860).

Second, we conducted a cloze task, presenting participants with our stimuli sentences without the object noun, and asking to complete them. Seventeen German native speakers took part (18–55 years of age). The results showed that in the context of *spill*,* water* was more predictable (cloze probability of 13.67%) than *ice cream* (cloze probability of 0.16%).^4^

In addition to the linguistic stimuli, the experiment presented visual displays with four concrete objects.^5^ Two of the four objects fit the category introduced by the restrictive verb (*spill*:* water*,* ice cream*), while all four fit the non‐restrictive verb (*order*:* water*,* ice cream*,* suitcase*,* coat*). The position of target and competitor objects was rotated to all possible positions (individual and mutual).

As mentioned, the referent noun was always fitting with the previous linguistic context and the gaze cue was always congruent, that is, cuing the object that is about to be mentioned. The main manipulation of the study was the presence of the gaze cue introduced before the target object was referred to linguistically.

**Figure 3:**
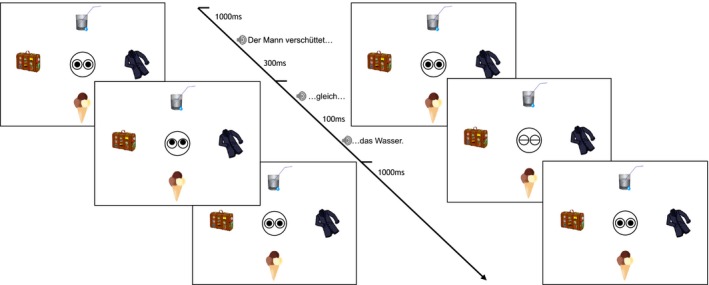
Exp. 1—Trial timeline example: Referent gaze condition (left) and no‐gaze condition (right).

Fig. 3 presents a trial timeline. The left side of the figure illustrates the referent gaze condition and the right side the no‐gaze condition. The sentence and the timeline are given in the middle, since they are identical for both gaze conditions. The visual scene with open eyes was presented 1,000 ms prior to sentence onset. The gaze cue (or closed eyes) was introduced 300 ms after the verb offset, that is, from adverb onset, to sentence end. Finally, after the end of the audio presentation, the eyes would look straight for another 1,000 ms.

In the no‐gaze condition, the eyes close in the relevant time window since we wanted to avoid introducing any additional information, but introduce the same amount of change in the visual context as present in the referent gaze condition.

#### 2.1.3 Fillers

Fillers included the same visual setting but differed in sentence structure and complexity of the linguistic stimulus, as well as the number of objects that fit the verb category. For instance, the sentence: *Bei der Großmutter gibt es immer leckere und hausgemachte Nudeln* (literal translation: *At grandma's there is always tasty and homemade pasta*) was presented with images of a chocolate, a cookie, a muffin, and a plate of pasta.

Thirty fillers were used, 25 of which had the opposite gaze condition as the items (5 had the same). In total, we created a ratio of 1:1 for gaze and no‐gaze conditions during the experimental session. Nineteen fillers were followed by simple yes/no comprehension questions that were answered by a key‐press. The questions were related exclusively to the linguistic content. This was done in order not to inspire extensive inspection of the visual scene, but rather so that participants consider the scene only optionally and freely in addition to the linguistic information.

A Latin square design was used in order to create four lists with 20 item and 30 filler trials each. Each list included each item in only one of the four linguistic conditions. The trials were pseudo‐randomized manually and an additional four lists were created with the opposite order of presentation. However, 32 participants (one half of the total number) saw the items in the referent gaze condition, while the other half of the participants saw them in the no‐gaze condition.

#### 2.1.4 Procedure

We used an EyeLink II head‐mounted eye tracker (SR Research, Ltd.; Mississauga, Ont., Canada) and tracked both eyes at a sampling rate of 250 Hz.^6^ The tracker was manually adjusted, calibrated, and validated using a 9‐point fixation stimulus. Participants were instructed to listen carefully to the sentences while looking freely at the presented scenes. They would advance the experiment by a key press after each item. This allowed them to take a break whenever they needed one. Two additional keys were used to answer the yes/no comprehension questions. A practice session of three trials preceded the experimental part. After having familiarized themselves with the experimental setup, the participants would continue to the experimental session by pressing a key on the keyboard. The experiment lasted for approximately 15 min.

#### 2.1.5 Variable coding and data analysis

First, in order to gain insight into the patterns of visual attention, we considered the proportion of fixations to the presented objects throughout a trial. Each of the presented objects is treated as a separate area of interest (AoI), with the gaze cue being an additional, fifth area.

Second, in order to statistically assess shifts in visual attention resulting from either verb constraints or gaze‐following, we analyzed new inspections, that is, the first of potentially a series of consecutive inspections of an AoI that occurred during a region of interest. We consider new inspections in two regions: (a) Verb region of interest: showing how the verb category influenced visual attention, hinting at an object being considered as most fitting to the verb constraint; and (b) Gaze region of interest: showing if the gaze cue inspired an immediate shift in visual attention.

Finally, the ICA events were extracted from the pupil jitter, summed over a duration of a relevant time window and statistically analyzed. For the ICA analysis, two time windows were of relevance: (a) Gaze time window: 600 ms from the onset of the gaze cue, that is, while the adverb was heard, showing the cost of considering the gaze cue; and (b) Reference time window: 600 ms from the middle of the referent noun, showing the cost of processing the linguistic reference.

In their VWP experiment, Demberg and Sayeed (2016) establish a time window taken from 600 to 1,200 ms from the onset of the critical word to be an appropriate window size and timing for the analysis of the ICA events. Since our critical words differ in length across items and since there is article variation among items, we corrected this potential confound by taking a time window that starts from the middle of the noun and considers the following 600 ms.

The middle of the referent noun was calculated by taking the audio duration of the whole word and using its half as the starting point, for each word individually.^7^ In addition, we analyzed the gaze window: 600 ms from the gaze cue onset.

The ICA events were extracted using the EyeWorks Workload Module software (version 3.12) for both eyes separately. Since there is no clear theoretical reason why differences should be expected for the two eyes, we combined the two datasets by summing the ICA events for corresponding time windows and conduct the analyses on the combined data.

All independent variables were contrast coded for the statistical analysis. New inspections, a binary dependent variable, required the use of generalized mixed effects models of binomial type. The analysis of the ICA, a count variable, required the use of generalized mixed effects models with Poisson distribution. All models included maximal converging random structure justified by the experimental design (Barr et al., 2013). The analyses were conducted in the R programming environment (version 3.4.0; R Core Team, 2017) using the *lme4* package (Bates, Mächler, Bolker, & Walker, 2015).

### 2.2 Results

#### 2.2.1 Proportion of fixations

**Figure 4:**
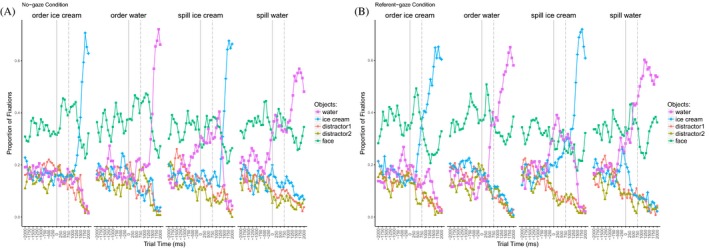
Exp. 1—Proportion of fixations aligned to the gaze cue onset (solid line). The dashed line presents article onset of the object noun phrase. (a) Four linguistic conditions of the no‐gaze condition. (b) Four linguistic conditions of the referent gaze condition.

Fig. 4 illustrates the proportions of fixations to all AoIs during a trial. Four linguistic conditions are presented both without the gaze cue (left‐hand side) and in the referent gaze condition (right‐hand side). The plots are aligned to the gaze cue onset, represented by the solid line. Note that the eyes would close at this point in the no‐gaze condition. In addition, the dashed line presents the onset of the article from the object noun phrase.

It is evident that the restrictive verb (*spill*) shifted the focus of visual attention to one particular object (*water*). The less fitting object (*ice cream*) was considered only upon being referred to linguistically (no‐gaze condition), or earlier, at the point of the gaze cue (referent gaze condition). In the context of the non‐restrictive verb (*order*), no preference for any of the objects was recorded until one of them was either referred to linguistically, or earlier, when it was gazed at. Thus, we found evidence that the visual attention is not only influenced by language, that is, the objects’ fit with the verb category, but also by the gaze cue.

#### 2.2.2 New inspections

We considered the probability of an inspection to fall in an AoI in a region of interest (from verb onset; from gaze cue onset). All fixations that fell within one AoI and prior to a fixation outside of that AoI were grouped together and considered as one inspection. For the statistical analysis we treated new inspections as a binary variable, since trials with a new inspection to the AoI were given the value “1” and those without a “0.”

First, we conducted statistical analysis of new inspections of an object (*water*,* ice cream*, distractors) in the verb region of interest (200 ms from verb onset to gaze onset). Second, we considered new inspections of *water* and *ice cream*, in the gaze region of interest (to article onset). The main models and collected results are given in Table 1. Detailed information about the results of the further comparisons is given in Table A1 (Appendix A).^8^

**Table 1:** Exp. 1—Results of the main models fitted for the new inspections analysis for both verb and gaze regions of interest

	(a) *water* inspections				(b) *ice cream* inspections				(c) distractors inspections			
Predictor	β	*SE*	*z*	*p*	β	*SE*	*z*	*p*	β	*SE*	*z*	
1. Verb region of interest												
Intercept	−1.928	0.065	−29.704	<2e−16***	−2.050	0.068	−29.97	<2e−16***	−1.446	0.074	−19.567	<2e−16***
Constraint	−0.458	0.104	−4.406	1.05e−05***	−0.168	0.114	−1.48	0.139	0.433	0.095	4.578	4.69e−06***

	(a) *water* inspections				(b) *ice cream* inspections			
Predictor	β	*SE*	*z*	*p*	β	*SE*	*z*	*p*
2. Gaze region of interest								
Intercept	−1.820	0.063	−28.788	<2e−16***	−2.239	0.085	−26.331	<2e−16***
Gaze	0.265	0.123	2.155	0.031*	0.305	0.154	1.987	0.047*
Plausibility	−0.454	0.133	−3.420	0.001***	0.972	0.172	5.663	1.48e−08***
Gaze:Plaus	−0.835	0.266	−3.143	0.002**	0.817	0.316	2.587	0.010**

*Notes*: *p < .05, **p < .01, ***p < .001.

1(a) WaterInsp ∼ Constraint + (1 + Constraint ‖ Subject) + (1 + Constraint ‖ Item), family = “binomial.”

1(b) IceInsp ∼ Constraint + (1 + Constraint | Subject) + (1 + Constraint | Item), family = “binomial.”

1(c) DistractInsp ∼ Constraint + (1 + Constraint|Subject) + (1 + Constraint | Item), family = “binomial.”

2(a) WaterInsp ∼ Gaze × Plausibility + (1 + Gaze × Plausibility ‖ Subject) + (1 + Plausibility ‖ Item), family = “binomial.”

2(b) IceInsp ∼ Gaze × Plausibility + (1 + Gaze × Plausibility ‖ Subject) + (1 + Plausibility ‖ Item), family = “binomial.”

Considering the verb region of interest we ran separate models for the inspections of relevant objects. The analysis of target inspections (*water*) showed a main effect of Constraint (*p* < .001) suggesting that more new inspections of *water* occurred upon hearing *spill* (*M* = 0.156, *SD* = 0.363) than *order* (*M* = 0.105, *SD* = 0.307). Competitor inspections (*ice cream*) showed no such effect (*p* = .139) suggesting that *ice cream* was looked at with no significant difference in the contexts of the two verbs. Finally, distractor inspections show a main effect of Constraint (*p* < .001), which suggests that there were more new inspections of the two distractors in the context of *order* (*M* = 0.234, *SD* = 0.428) than *spill* (*M* = 0.169, *SD* = 0.375).

Furthermore, we considered the gaze region of interest, showing a shift in attention inspired by the gaze cue. The model including new inspections of *water* reveals main effects of Gaze (*p* = .031), Plausibility (*p* = .001), and most important, the Gaze:Plausibility interaction (*p* = .002). Further comparisons show that in the subset of the plausible target *water*, there is a main effect of Gaze (β = 0.648, *SE* = 0.178, *z* = 3.641, *p* < .001), while in the subset of the possible target *ice cream* there was no such effect (*p* = .415), suggesting that *water* was inspected more when cued at (ref. gaze: *M* = 0.224, *SD* = 0.417 vs. no‐gaze: *M* = 0.128, *SD* = 0.334).

The model with new inspections to *ice cream* also showed main effects of Gaze (*p* = .047), Plausibility (*p* < .001), and a Gaze:Plausibility interaction (*p* = .010). Further comparisons show that in the subset of *water* there was no effect of Gaze (*p* = .688). Such an effect was present in the *ice cream* subset (β = 0.721, *SE* = 0.187, *z* = 3.853, *p* < .001), suggesting that *ice cream* was more readily inspected when it was gazed at (ref. gaze: *M* = 0.203, *SD* = 0.402 vs. no‐gaze *M* = 0.112, *SD* = 0.315).

#### 2.2.3 The Index of Cognitive Activity

**Figure 5:**
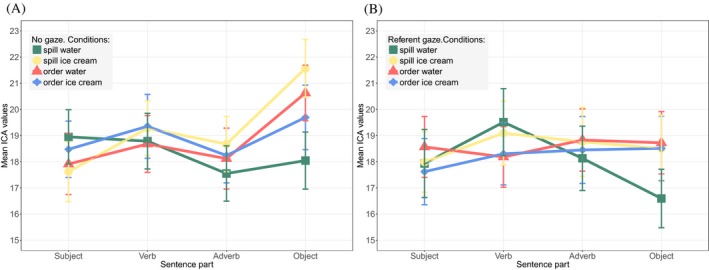
Exp. 1—Mean ICA values at the four time windows of a sentence. Points marked as *Adverb* (Gaze time window) and *Object* (Reference time window) are relevant for the analysis (95% CI error bars). (a) No‐gaze condition. (b) Referent gaze condition.

The ICA findings are illustrated by Fig. 5. We present the no‐gaze condition on the left‐hand side and the referent gaze condition on the right‐hand side. The x‐axis presents the 600 ms time windows for the four sentence parts. The two time windows relevant to the analysis are the points labeled as *Adverb* (Gaze time window), and *Object* (Reference window). Each point on the graph represents the mean value of the ICA events per condition for a 600 ms time window. We clearly observe no difference at the point of the Adverb, while significant differences exist at the point of the Object both among the linguistic conditions and between the two gaze conditions. The main models and collected results are given in Table 2. Detailed information about the results of the further comparisons is given in Table A2 (Appendix A).

**Table 2:** Exp. 1—Results of the main models fitted for the ICA analysis

	(a) Gaze time window				(b) Reference time window			
Predictor	β	*SE*	*z*	*p*	β	*SE*	*z*	*p*
Intercept	2.881	0.029	98.03	<2e−16***	2.915	0.026	110.29	<2e−16***
Gaze	0.005	0.055	0.09	0.925	−0.113	0.051	−2.21	0.027*
Constraint	0.004	0.016	0.25	0.802	0.038	0.024	1.59	0.111
Plausibility	0.017	0.018	0.97	0.332	0.058	0.027	2.11	0.034*
Const:Plaus	−0.060	0.026	−2.28	0.021*	−0.184	0.042	−4.34	1.4e−05***
Half	−0.031	0.0134	−2.31	0.016*	−0.052	0.022	−2.39	0.017*
Half:Gaze	—	—	—	—	−0.027	0.043	−0.61	0.539

*Notes*: *p < .05, ***p < .001.

(a) ICA ∼ Const × Plaus + Half + Gaze + (1 + Const + Plaus ‖ Subject) + (1 + Const + Plaus ‖ Item), family = Poisson (link = “log”).

(b) ICA ∼ Const × Plaus + Half × Gaze + (1 + Const × Plaus ‖ Subject) + (1 + Const × Plaus ‖ Item), family = Poisson (link = “log”).

The analysis of the gaze time window revealed a main effect of Half^9^ (*p* = .016) and a Constraint:Plausibility interaction (*p* = .021). However, further comparisons show that this interaction is carried by the opposite trend of the two Plausibility levels in the subsets of the two verbs, that remain not close to statistical significance (subset *spill*:* p* = .104; subset *order*:* p* = .624).

Considering the reference window, we found a main effect of Gaze on cognitive load (*p* = .027), suggesting that the presence of the gaze cue led to the reduction of load on the subsequent reference (no‐gaze: *M* = 19.983, *SD* = 7.296 vs. referent gaze: *M* = 18.08, *SD* = 7.723). Moreover, we found a significant Constraint:Plausibility interaction (*p* < .001), as well as a main effect of Plausibility on cognitive load (*p* = .034). Further comparisons show a main effect of Plausibility in the subset of *spill* (β = 0.152, *SE* = 0.035, *z* = 4.37, *p* < .001), suggesting that *spill water* (*M* = 17.319, *SD* = 7.102) induced less cognitive load than *spill ice cream* (*M* = 20.041, *SD* = 7.746). No such effect was found in the non‐constraining subset (*p* = .249), suggesting no difference between *order water* and *order ice cream*. Finally, we found no Half:Gaze interaction (*p* = .539), but a main effect of experimental Half on cognitive load (*p* = .017), since the first half of the experiment induced higher load (*M* = 19.516, *SD* = 7.553) than the second half (*M* = 18.599, *SD* = 7.563).

### 2.3 Discussion

The eye movement data suggest that the selectional preferences of the restrictive verb *spill* inspired creating a clear prediction for *water* to be mentioned in the continuation of the sentence. Consequently, *water* was easier to process in the context of *spill* (in comparison to *order*). Also, in the context of the verb *spill*,* water* proved to be easier to process than *ice cream*. Importantly, with the introduction of the gaze cue, this effect of verb‐based anticipations changed. The gaze cue towards an object led to the inspection of the cued object, even when the verb selectional preferences did not previously put it in the focus of visual attention. That is, listeners followed the gaze cue to inspect the ice cream after hearing *spills* when otherwise the water would have been inspected, or after *order* when any of the four objects were equally expected. Consequently, the presence of the gaze cue led to the reduction of cognitive load on the referent noun no matter how constraining the verb or how good the fit with the noun was. These findings are in line with previous literature (Hanna & Brennan, 2007; Knoeferle & Kreysa, 2012; Macdonald & Tatler, 2013, 2014; Staudte & Crocker, 2011; Staudte et al., 2014) and further support it by reporting immediate cognitive effort required for processing a reference.

In addition, we were able to expand the current understanding of gaze perception by assessing immediate cognitive load it induces. Interestingly, even though it created a shift in visual attention to an object often not previously considered, this did not induce immediate cognitive load. Moreover, we expected the reduction of cognitive load on the referent noun to be preceded by an increased cost at the point of the gaze cue—to compensate for the lower effort on the reference. Such a distribution of cognitive load was not found, since no additional effort was measured on the gaze cue.

## 3 Experiment 2

This experiment challenged the conclusions from the first study by employing a mismatching gaze cue. In Experiment 1, we compared a plausible referent with a possible one (low plausibility), which induced differences in cognitive load on the linguistic reference, but not on the gaze cue itself. The present study builds on these findings by exaggerating the difference between the two conditions and employing an impossible object that doesn’t fit the linguistic context. First, we examined whether the gaze cue helps reduce cognitive load on the linguistic reference even when they do not fit the previous context, and, second, whether the cue to such an object is more costly, since it violates the context.

We aimed to answer the following research questions:

a) How does a gaze cue affect cognitive load on the following reference when the cued object does not fit the previous linguistic context?
a) Is the gaze cue to a mismatching object costly?

We expected mismatching gaze to be surprising, and thus in itself more costly. Consequently, though, this was expected to lead to a reduction in cognitive load on the corresponding linguistic reference.

### 3.1 Method

The experiment made use of 2 × 2 experimental design, combining Gaze (no‐gaze vs. referent gaze) and referent noun Fit (fitting vs. mismatching). Only restrictive verbs were used (*spill*), combined with either a thematically fitting (*water*) or mismatching referent noun (*sausage*).

#### 3.1.1 Participants

Thirty‐six participants (23 female), students at Saarland University, took part in the study and were monetarily reimbursed for their participation. None of the participants took part in or were familiar with Experiment 1. Their ages ranged from 18 to 34 years (*M* = 23.36). Two students were excluded from the analysis due to technical issues, and two others because their mother tongue was established to be Luxemburgish. Thus, the data from 32 participants, all German native speakers, were analyzed.

#### 3.1.2 Items

The experiment made use of 20 item trials. The sentence structure was the same as in Experiment 1. This time, however, only the restrictive verbs were used (*spill*). The linguistic manipulation considered the Fit of the referent noun to the category introduced by the verb. The referent could be fitting (*spill water*) or mismatching (*spill sausage*). In order to counterbalance the referent nouns, the items were run in two versions. Version A included the verb fitting one noun in the item (*spill water* vs. *sausage*), while the verb in version B fit the other noun (*grill sausage* vs. *water*).^10^

Note that the gaze cue was, again, always congruent (cuing the object subsequently referred to); however, its fit with the linguistic context depended on the fit of the referent noun. When the referent noun did not fit the verb, that made the gaze cue to the object in question mismatching as well.

**Figure 6:**
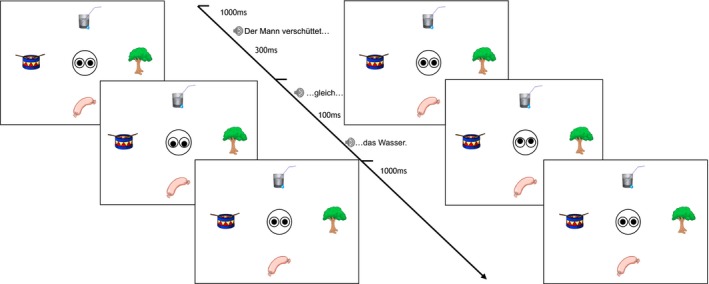
Exp. 2—Trial timeline example: Mismatching condition (left) and fitting condition (right).

Visual scenes are of the same structure as in Experiment 1, but now only one presented object fits the verb category. A trial timeline is illustrated in Fig. 6, showing the referent gaze condition. On the left side of the figure is the mismatching condition, while the right side illustrates the fitting condition. Note that the no‐gaze condition was also part of the experimental manipulation. As previously, the position of target and competitor objects was rotated to all possible positions (individual and mutual).^11^

#### 3.1.3 Fillers

During the experiment, participants were presented with 75 trials in total, 55 of which were fillers. The fillers, again, differed from the experimental items in the complexity of the sentence structure and the number of presented objects that fit the verb category. For instance, the sentence: *Immer noch trägt der Vater seine Armbanduhr* (literal translation: *Still wears the father his watch*) presented with the images of a watch, a coat, a pocket flashlight, and a clock. Thirty‐five filler trials were followed by a simple yes/no comprehension question referring only to the content of the heard sentence.

The gaze cue was present in two‐thirds of all trials (10 items, 40 fillers). Ten percent of the total number of trials included anomalous sentences (10 items, 5 fillers). Only 16% of all trials included an anomalous gaze cue, that is, gaze that was cueing a mismatching object (5 items, 3 fillers).

The Latin square design was used to create four lists with one condition per item each. The trials were pseudo‐randomized manually, and an additional four lists were created with the opposite trial order.^12^ The experimental procedure was the same as in Experiment 1. The duration of the experiment was approximately 20 min.

### 3.2 Results

The same measures were employed, and the same analyses conducted as in Experiment 1, except for the new inspections analysis, where only the gaze region of interest was considered, due to the differing experimental design of Experiment 2. Again, all independent variables were contrast coded for the statistical analyses, and all models included maximal converging random structure justified by the experimental design (Barr et al., 2013).

#### 3.2.1 Proportion of fixations

**Figure 7:**
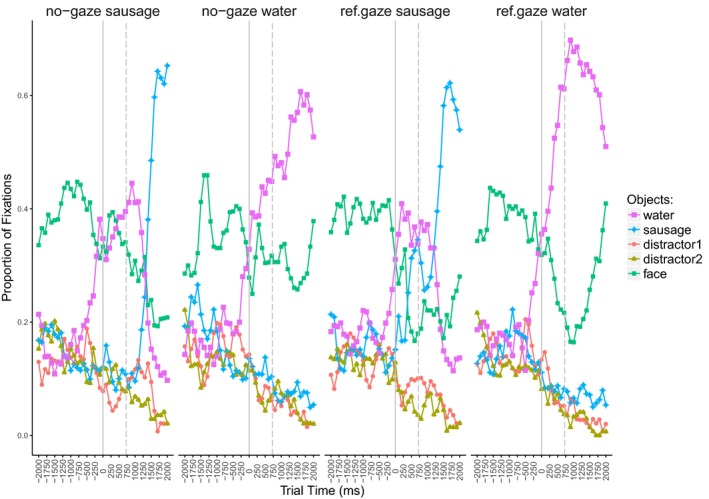
Exp. 2—Proportion of fixations to presented objects in the four experimental conditions, aligned to the gaze cue onset (solid line). The dashed line marks the onset of the article from the object noun phrase.

Fig. 7 shows the proportion of fixations to all presented objects during a trial. As previously, the plots are aligned to the onset of the gaze cue (marked with the solid line). In addition, the dashed line presents the onset of the article from the object noun phrase.

The verb (*spill)* shifts the focus of visual attention to one particular object (*water*). The mismatching object (*sausage*) is considered only upon being referred to linguistically (no‐gaze condition), or earlier, at the point of the gaze cue (referent gaze condition). Thus, we observe the same pattern as in Experiment 1, namely, of the gaze cue shifting visual attention, on a par with the linguistic information.

#### 3.2.2 New inspections

Because of the unusual gaze cue (to *sausage*), which was, however, entirely congruent with the subsequent noun, we hypothesized that participants might get used to this over time and that this could change their perception and utilization of the cue. Hence, we include an additional variable in the analysis, namely, experiment Half, which codes the information of the experiment part in which a participant was presented with a given trial. Table 3 presents the main models and collected results. Detailed information about the results of the further comparisons is given in Table A3 (Appendix A).^13^

**Table 3:** Exp. 2—Results of the main models fitted for the new inspections analysis (gaze region of interest)

	(a) *water* inspections				(b) *sausage* inspections			
Predictor	β	*SE*	*z*	*p*	β	*SE*	*z*	*p*
Intercept	−1.617	0.098	−16.581	<2e−16***	−2.434	0.126	−19.299	<2e−16***
Gaze	0.129	0.156	0.826	0.409	0.555	0.224	2.476	0.013*
Fit	−0.562	0.162	−3.467	0.001***	0.678	0.218	3.107	0.002**
Gaze:Fit	−0.409	0.313	−1.308	0.191	1.199	0.436	2.749	0.006**
Half	0.138	0.155	0.890	0.373	−0.477	0.214	−2.226	0.026*
Half:Gaze	−0.005	0.310	−0.017	0.986	0.343	0.429	0.800	0.424

*Notes*: *p < .05, **p < .01, ***p < .001.

(a) WaterInsp ∼ Gaze × Fit + Half × Gaze + (1 + Fit ‖ Subject) + (1 + Fit ‖ Item), family = “binomial.”

(b) SausageInsp ∼ Gaze × Fit + Half × Gaze + (1 + Gaze + Fit ‖ Subject) + (1 + Fit  ‖ Item), family = “binomial.”

The model which included new inspections to *water* revealed only the main effect of Fit (*p* = .001), suggesting that *water* was inspected more in the *spill water* condition (*M* = 0.213, *SD* = 0.409) than in *spill sausage* (*M* = 0.133, *SD* = 0.34). The lack of a main effect of Gaze (*p* = .409) and Gaze:Fit interaction (*p* = .191) suggests that *water* inspired more new inspections regardless of what, if anything, was gazed at.

The model with new inspections to *sausage* showed a Gaze:Fit interaction (*p* = .006), and main effects of Gaze (*p* = .013), Fit (*p* = .002), and Half (*p* = .026). Further comparisons show that in the *no‐gaze* subset, there was no effect of Fit (*p* = .826), while such an effect was present in the *referent gaze* subset (β = 1.288, *SE* = 0.279, *z* = 4.618, *p* < .001), suggesting that *sausage* was more readily inspected only when it was gazed at (ref. gaze: *M* = 0.123, *SD* = 0.329 vs. no‐gaze: *M* = 0.067, *SD* = 0.249). Finally, the main effect of Half in the full model suggests that more new inspections of *sausage* were initiated in the first part of the experiment (1st half: *M* = 0.113, *SD* = 0.317 vs. 2nd half: *M* = 0.082, *SD* = 0.274).

In sum, new inspections of *water* were more likely both when there was no gaze cue, and when there was a referent gaze to *water*, while *sausage* was more likely to attract new inspections only when it was gazed at.^14^ Considering the two halves of the experiment, the only observed difference lies in the new inspections of *sausage*; namely, there were more new inspections of *sausage* in the first part.^15^

#### 3.2.3 The Index of Cognitive Activity

Table 4 presents the main models and collected results. Detailed information about the results of the further comparisons is given in Table A4 (Appendix A).

**Table 4:** Exp. 2—Results of the main models fitted for the ICA analysis

	(a) Gaze time window				(b) Reference time window			
Predictor	β	*SE*	*z*	*p*	β	*SE*	*z*	*p*
Intercept	2.773	0.038	72.90	<2e−16***	2.782	0.036	76.32	<2e−16***
Gaze	0.062	0.034	1.86	0.063 .	−0.020	0.034	−0.58	0.562
Fit	0.037	0.034	1.09	0.275	0.222	0.045	4.94	7.76e−07***
Gaze:Fit	0.092	0.054	1.70	0.090 .	−0.022	0.050	−0.44	0.664
Half	−0.040	0.039	−1.03	0.304	−0.052	0.035	−1.51	0.131
Half:Gaze	0.069	0.059	1.16	0.246	−0.131	0.061	−2.17	0.030*

*Notes*: .*p* < .1,*p < .05, **p < .01, ***p < .001.

(a) ICA ∼ Gaze × Fit + Half × Gaze + (1 + Gaze × Fit + Half × Gaze ‖ Subject) + (1 + Fit | Item), family = Poisson (link = “log”).

(b) ICA ∼ Gaze × Fit + Half × Gaze + (1 + Gaze × Fit + Half × Gaze ‖ Subject) + (1 + Fit | Item), family = Poisson (link = “log”).

We first considered the reference time window and found a main effect of Fit on cognitive load (*p* < .001), suggesting that the anomalous *spill sausage* required more load (*M* = 18.410, *SD* = 6.983) than *spill water* (*M* = 14.959, *SD* = 6.928). Considering the effect of the gaze cue on the cost of the referent, a Half:Gaze interaction was observed (*p* = .030). Further analysis showed a marginal main effect of Gaze in the second half of the experiment (β = −0.091, *SE* = 0.047, *z* = −1.93, *p* = .054), suggesting that the referent gaze reduced cognitive load on the referent noun in both linguistic conditions (ref. gaze: *M* = 15.692, *SD* = 6.804 vs. no‐gaze: *M* = 16.987, *SD* = 7.631). No such effect was found in the first half (*p* = .392).

**Figure 8:**
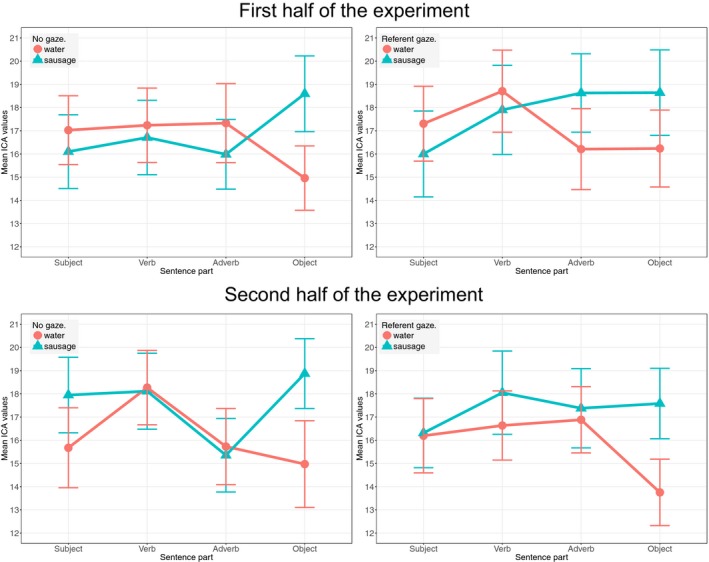
Exp. 2—Mean ICA values in the four time windows of a sentence in the first (above) and the second (below) half of the experiment, and the no‐gaze (left) and referent gaze (right) conditions. Points marked as *Adverb* (Gaze time window) and *Object* (Reference time window) are relevant for the analysis (95% CI error bars).

Since gaze affected referent processing in each experimental half differently, we also considered cognitive load on the cue itself (gaze window) for each experimental half separately. The first part of the experiment revealed a Gaze:Fit interaction (β = 0.179, *SE* = 0.084, *z* = 2.13, *p* = .033). Further comparisons required to interpret the interaction are not conducted due to power issues with too‐small subsets. Nevertheless, Fig. 8 shows that the interaction is the result of the cognitive load induced by the anomalous gaze cue to *sausage* being higher than that resulting from the gaze cue to *water*, while such a difference between the two conditions is non‐existent in the no‐gaze condition. Finally, in the second half, we found a main effect of Gaze (β = 0.099, *SE* = 0.045, *z* = 2.20, *p* = .028), suggesting that the referent gaze induced higher cognitive load (*M* = 17, *SD* = 6.971) than the no‐gaze condition (*M* = 15.536, *SD* = 7.058).

Fig. 8 illustrates the results. The top two plots show the data in the first part of the experiment, while the lower two illustrate the second experiment half. The left‐hand side shows the no‐gaze condition, and the right‐hand side the referent gaze condition. As in Fig. 5, the Adverb point represents the gaze time window, and the Object point the reference time window. The pattern in the no‐gaze condition did not change in the two experimental halves, while the referent gaze condition yielded significantly different effects. We see that in the first part the anomalous gaze cue was inducing more cognitive load than the congruent referent gaze. In the second half, though, the gaze cue became more costly in general. As for the cost of the referent noun, initially, the existence of the gaze cue did not reduce cognitive load either on the congruent or the incongruent noun. In the second half, however, we observe a marginal facilitation effect caused by the previous gaze cue for the processing of both congruent and incongruent references.

### 3.3 Discussion

The eye movement data show that the linguistic context inspired clear prediction of only one of the depicted objects (*water*). In addition, we see evidence of gaze following, even when the cued object was not only unpredicted, but worse, not fitting the verb (*sausage*). In the case of mismatching gaze cue, the cued object was inspected the most (*sausage*); however, the fitting and preferred object (*water*) was nevertheless not discarded and still inspected more than the two distractor objects. This is particularly evident in the second part of the experiment, where the mismatching gaze cue inspired equal inspection of both cued and predicted objects, suggesting a slight change in gaze cue following that occurred during the experiment.

Considering the cognitive load results, initially, the existence of gaze did not have an effect on the cost of processing the referent noun. No benefit of the gaze cue was found even in the fitting referent gaze condition, where the predicted object (*water*) was cued. Regarding the effort required on the gaze cue, the load induced by the mismatching cue itself was higher than that induced in both fitting gaze and no‐gaze conditions. In the second half of the experiment, cognitive load on the referent noun was marginally reduced due to the gaze cue; while the cue itself (to both fitting and mismatching object) now induced higher cognitive effort.

We understand that participants gradually started adapting to, and relying on, the surprising gaze cue. Initially, the mismatching gaze cue caused “concern” that anomalies are possible, not only at the point of the gaze, but potentially also later on the reference, resulting in no reduction of cognitive load on the referent noun. Over time, gaze proved to always be congruent with the referent noun. Simultaneously, the cognitive load on the gaze cue rose. Since gaze reliably gave away reference information, this led to more alertness on the cue (increasing the load on the cue in general) and inspired making use of its informativity (slightly lowering the load on the reference). The anomalous condition proved to be costly. However, in the presence of the gaze cue, we observed a tendency for facilitated processing of the subsequent anomalous reference.

Comparing the results obtained from eye movement and cognitive load analyses, we see that gaze was followed and induced a shift in visual attention throughout the experiment, even though the cognitive load results suggest that it was initially not exploited to the same extent. Higher cognitive effort induced by the anomalous gaze cue in the first experiment half was paired with new inspections of both the cued (*sausage*) and the anticipated object (*water*), where more inspections were directed to the cued object. At the same time, no increase in load on the fitting gaze cue was paired with new inspections of only that object. In the second half of the experiment, however, the cognitive load increased on both types of referent gaze, while the new inspections still patterned differently. The fitting gaze cue led to, again, new inspections to only the anticipated object (*water*), while the mismatching gaze cue equally inspired inspections to both the cued (*sausage*) and the anticipated object (*water*).

## 4 Experiment 3

Manipulating the reliability of gaze and language while giving equal prominence to both cues, Macdonald and Tatler (2014) found that language was the most disruptive cue in their study, when least reliable. Thus, they concluded that language is the dominant cue, preferred over gaze. In addition, helpful gaze benefited performance by speeding up both first fixations to the target, and reaction times on the task. When language was 100% accurate, the gaze cue was superfluous, albeit still followed, and when incongruent with language, it would slow down performance. In sum, gaze was found to be disruptive when incongruent to more informative language, but only when the cue was established to be reliable.

Again, measuring immediate cognitive load, we follow up on our previous findings and aim to answer the following research question:

b) Would a gaze cue, fitting the previous linguistic context but incongruent with the following reference, affect the cognitive load on the (also fitting) referent noun?

**Figure 9:**
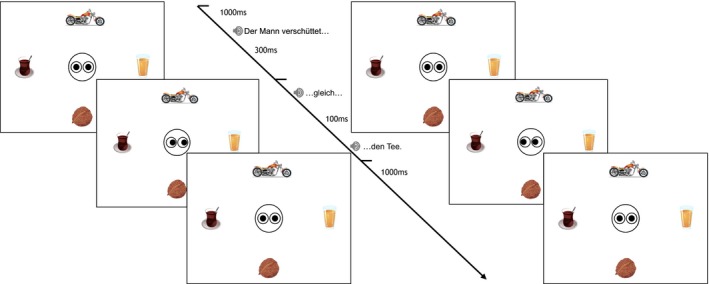
Exp. 3—Trial timeline example: Competitor gaze condition (left) and target gaze condition (right).

As illustrated in Fig. 9, participants were presented with four objects, two of which were of **equally** good fit with the verb category (*tea* and *juice*). Thus, anticipation for either of those two objects (gazed at vs. referred to) was to be created prior to the gaze cue. The main manipulation was the incongruent gaze cue, that is, cuing one fitting object (*juice*) while the other one is subsequently referred to (*tea*).

Two developments could be expected from this experimental setup. The gaze cue could be automatically followed, as was the case in the previous two experiments, and thus, create expectation for the cued object. Subsequently, when the other fitting object is actually referred to, this would lead to an increase in cognitive load at the referent noun (otherwise fitting the context, and not surprising in itself). Alternatively, the incongruent gaze cue might not induce cost on the referent noun, since no clear expectation was created before the gaze cue (two equally probable target objects presented), and since the gaze cue is sometimes incongruent throughout the experiment (15% of trials), it could be equally acceptable to have either the cued object or the other fitting object complete the sentence.

Macdonald and Tatler (2014) found that attentional effects of gaze are affected by its reliability. Thus, we made sure that the gaze cue was overall reliable (85% congruent with the reference) in the context of the experiment.

### 4.1 Methods

One independent variable with three levels was created, namely Gaze (no‐gaze vs. target gaze vs. competitor gaze). The linguistic reference was always plausible and fitting with the previous context; however, the previous gaze cue was not always cuing the target object but was sometimes incongruent (i.e., cuing the competitor).

#### 4.1.1 Participants

33 Saarland University students took part in this study (24 women) and were monetarily reimbursed for their participation. Their ages ranged from 18 to 39 years (*M* = 23.52). Participants were all native speakers of the German language with normal or corrected‐to‐normal vision. None of the participants took part in or were familiar with either Experiment 1 or Experiment 2.

#### 4.1.2 Items

We created 21 item trials. They included the same sentence structure as was the case in the previous two studies. As in Experiment 2, only restrictive verbs were used (*spill*). The referent noun always fitted with the previous linguistic context (*spill*:* tea* or *juice*).^16^

The visual scenes were of the same structure as in the previous two experiments.^17^ Of the four presented objects, two did not fit the verb category (distractors: *motorbike*,* walnut*), while the other two were equally plausible in the context of the given verb (*spill*:* tea*,* juice*). Again, the position of target and competitor objects was rotated to all possible (individual and mutual) positions.

A pretest was conducted in which the displays were presented together with the beginning of the stimulus sentence: *The man spills now ..*.. Written instructions read that the sentence should fit the visual display, and the participants’ task was to complete the sentence, by naming the object they would expect at the end of the sentence. Based on the results collected from 36 German native speakers, we chose 21 sentence and scene pairs where both potential target objects were equally often selected. In this way, we created 21 item trials that included visual scenes with two objects being equally plausible in the given linguistic and visual context.

As mentioned, the only experimental manipulation considered the gaze cue, which was either absent (no‐gaze), or cuing one of the two fitting objects, that is, either the target (congruent gaze) or the competitor (incongruent gaze). The experiment was run in two versions. Version A used one sentence continuation (*spill tea*), while in version B the other object was referred to (*spill juice*).

A trial timeline is illustrated in Fig. 9. The left‐hand side presents the competitor gaze condition, while the right‐hand side shows the target gaze condition. The sentence used and the timeline were identical for different gaze conditions.

#### 4.1.3 Fillers

The experiment included 100 trials in total, 79 of which were fillers. The gaze cue was present in two‐thirds of all trials (14 items, 53 fillers). Incongruent gaze made up only 10% of the overall number of trials, that is, 15% of the trials including a gaze cue (7 items, 3 fillers). Simple yes/no comprehension questions followed 57 of the filler trials. As was the case in previous experiments, filler trials differed from the experimental ones in terms of sentence structure and the number of objects that fit the verb selectional features. For instance, the sentence *Von allem Früchten mag der Bruder am liebsten Himbeeren* (literal translation: *Of all fruits likes the brother the most raspberries*) was presented with a display showing broccoli, a strawberry, a banana and a raspberry. Thus, three objects were potential targets in this example trial.

The experiment was preceded by a practice session that included three trials illustrating only no‐gaze and target gaze conditions (no incongruent gaze cue). Again, we used the Latin square design to create three lists where each item was presented in only one condition. The presentation of trials in a list was pseudo‐randomized manually, and an additional three lists were created with opposite trial order. Experimental procedure was the same as in the previous two experiments. The duration of the experiment was approximately 30 min.

### 4.2 Results

As in the previous two studies, we consider the following three measurements: (a) the proportion of fixations throughout a whole trial, (b) the statistical analysis of new inspections in a region of interest, and (c) the analysis of the ICA events in the gaze time window and the reference window. As done previously, all independent variables were contrast coded for the statistical analyses, and all models included maximal converging random structure justified by the experimental design (Barr et al., 2013).

#### 4.2.1 Proportion of fixations

**Figure 10:**
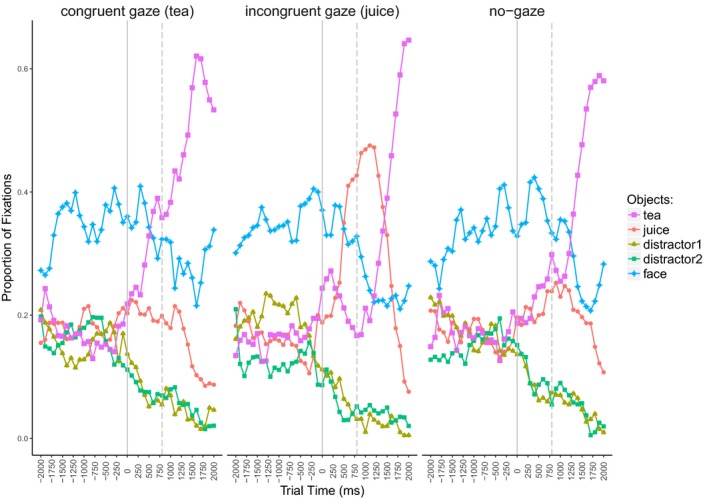
Exp. 3—Proportion of fixations to presented objects in the three conditions: congruent (target), incongruent (competitor) gaze, and no‐gaze. Gaze cue onset is marked with the solid line. The dashed line marks the onset of the article from the object noun phrase.

Fig. 10 illustrates the proportion of fixations to the four presented objects (and the face) during a trial. We show the fixation patterns for the three gaze conditions, namely, congruent gaze (*tea*), incongruent gaze (*juice*), and the no‐gaze condition. As previously, the plots are aligned to the onset of the gaze cue (closing of the eyes in the no‐gaze condition) which is marked with the solid line. The dashed line illustrates the onset of the article from the object noun phrase.

We see that the introduction of the gaze cue led to a shift in visual attention and the cued object became the most fixated one. However, at the same time, the other potential target was still considered, though to a lesser degree. In the no‐gaze condition, where no object was cued, we see that both potential target objects were inspected more than the distractors, but with no preference for one of the two. Finally, upon the referent noun onset, the most inspected object was the named one.

#### 4.2.2 New inspections

We considered new inspections in the gaze region of interest, in order to see how the gaze cue influenced the immediate inspection of the presented objects.^18^ We ran two different models, one for the target (*tea*) inspections and one for the inspections of the competitor (*juice*). The independent variable was contrast coded in such a way that NTgaze codes the comparison of no‐gaze and target gaze conditions; NCgaze—no‐gaze vs. competitor gaze, and TCgaze—target gaze vs. competitor gaze. Table 5 presents the fitted models and collected results.

**Table 5:** Exp. 3—Results of the two models fitted for the new inspections analysis (gaze region of interest)

	(a) Target inspections				(b) Competitor inspections			
Predictor	β	*SE*	*z*	*p*	β	*SE*	*z*	*p*
Intercept	−1.798	0.105	−17.071	<2e−16***	−1.773	0.090	−19.755	<2e−16***
NTgaze	−0.202	0.209	−0.966	0.334	0.127	0.214	0.591	0.554
TCgaze	−0.733	0.226	−3.253	0.001**	0.633	0.221	2.867	0.004**



*Notes*: **p < .01, ***p < .001. (a) TargetInsp ∼ NTgaze + TCgaze + (1 + NTgaze + TCgaze ‖ Subject) + (1 | Item), family = “binomial.”

(b) CompetitorInsp ∼ NCgaze + TCgaze + (1 + NCgaze + TCgaze ‖ Subject) + (1 | Item), family = “binomial.”

The model run on the target inspections revealed no effect of NTgaze (*p* = .334), that is, no difference between no‐gaze and the target gaze condition, but a main effect of TCgaze (*p* = .001), suggesting that there were more new inspections of the target (*tea*) when it was cued (*M* = 0.185, *SD* = 0.388) than when the competitor object (*juice*) was cued (*M* = 0.107, *SD* = 0.309).

The model run on the new inspections of the competitor showed, again, no difference between no‐gaze and competitor gaze conditions (*p* = .554), but a main effect of TCgaze (*p* = .004), suggesting more looking at *juice* when that object was also cued (*M* = 0.201, *SD* = 0.401) than when *water* was cued (*M* = 0.112, *SD* = 0.316).

#### 4.2.3 The Index of Cognitive Activity

**Figure 11:**
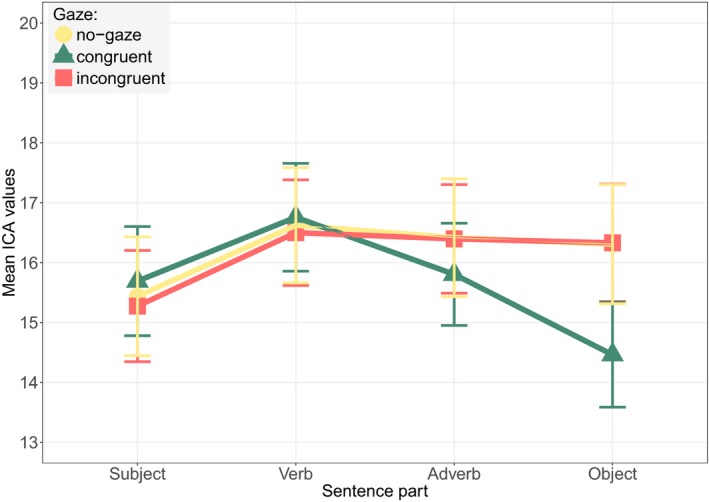
Exp. 3—mean ICA values in the four time windows of a sentence in no‐gaze, congruent (target), and incongruent (competitor) gaze conditions. Points marked as *Adverb* (Gaze time window) and *Object* (Reference time window) are relevant for the analysis (95% CI error bars).

As Fig. 11 shows, there is a difference in cognitive load on the referent noun induced by target gaze, in comparison to both no‐gaze and competitor gaze conditions. Since the incongruent and no‐gaze conditions show no difference between each other, we treat them together in the statistical analysis of the reference window and compare this to the congruent gaze condition.

Table 6 presents the fitted models and collected results. A main effect of Gaze on cognitive load (*p* = .006) suggests that the congruent gaze cue led to the reduction of load on the subsequent reference (no‐gaze: *M* = 16.307, *SD* = 7.678; incongruent gaze: *M* = 16.33, *SD* = 7.55; congruent gaze: *M* = 14.461, *SD* = 6.729). In addition, a main effect of experimental Half was found (*p* = .018), but no interaction (*p* = .778), since cognitive load was overall lower in the second part of the experiment (1st half: *M* = 16.548, *SD* = 7.374 vs. 2nd half: *M* = 14.925, *SD* = 7.297).

**Table 6:** Exp. 3—Results of the two models fitted for the ICA analysis. Note that the variable Gaze denotes different comparisons in the two models. In the gaze window, we compare the existence of the gaze cue: no‐gaze versus ref. gaze condition. In the reference window, the two conditions that behaved similarly are collapsed: congruent gaze versus no‐gaze & incongruent gaze

Predictor	(a) Gaze time window				(b) Reference time window			
	β	*SE*	*z*	*p*	β	*SE*	*z*	*p*
Intercept	2.758	0.035	77.92	<2e−16***	2.723	0.042	64.27	<2e−16***
Gaze	0.016	0.049	0.33	0.745	0.154	0.056	2.74	0.006**
Half	−0.063	0.034	−1.82	0.069 .	−0.082	0.035	−2.36	0.018*
Half:Gaze	−0.111	0.087	−1.28	0.201	−0.026	0.093	−0.28	0.778

*Notes*: .*p* < .1, *p < .05, **p < .01, ***p < .001.

(a) ICA ∼ Half × Gaze + (1 + Half × Gaze | Subject) + (1 | Item), family = Poisson (link = “log”).

(b) ICA ∼ Half × Gaze + (1 + Half × Gaze | Subject) + (1 | Item), family = Poisson (link = “log”).

In the Gaze window, we considered the two gaze conditions as one (target gaze and competitor gaze), since at the point of the gaze cue it is not known if the cue will end up being congruent or not; that is, we compared no‐gaze with the gaze cue. No significant differences were found.

### 4.3 Discussion

Without the gaze cue, the eye movement data revealed that both potential target objects are equally considered. Gaze shifts the visual attention to the cued object. However, the other potential target is not immediately discarded. Thus, we see an interplay of linguistic and visual cues. The verb activates two visually presented objects as potential referents for the following noun phrase. Gaze further highlights one of those objects; however, this does not lead to disregarding the other fitting object—rather, this object continues being activated. It is only upon hearing the referent noun that the non‐mentioned object is discarded.

Cognitive load results suggest that the incongruent gaze cue was not costly as such; rather, it induced the same effort as not having a cue. However, having a congruent (target) gaze cue has shown to be beneficial for the effort required to process the target noun. Again, we found evidence that the (fitting) congruent gaze cue facilitates reference processing without inducing cost in itself.

Unlike the present study, previous explorations into gaze congruency speak of a cost of incongruency. Incongruent gaze led to longer reaction times in behavioral tasks and is thus considered to be more costly than neutral or congruent gaze (Macdonald & Tatler, 2014; Staudte & Crocker, 2011). More recently, Jachmann et al. (2017) examined ERPs and similarly found a cost of an incongruent gaze cue. It is to be noted, however, that the design of the present experiment differs in important ways from the previous work.

First, we used full sentences where linguistic constraints inspired anticipation of only certain depicted objects. Macdonald and Tatler (2014) used no linguistic context, gaze being the only piece of information based on which a prediction for a specific object could have been made. When this cue was made reliable, it was followed and trusted, which led to disruption when it proved to be misleading. Similarly, Jachmann et al. (2017)'s gaze cue is the first piece of information based on which a prediction about the target can be made. Even though they use comparative sentences (*Compared to the car, the house is relatively small, I think*.), the gaze cue was presented before the second noun is uttered, hence, also prior to the comparative adjective. In our study, the linguistic context included a restrictive verb based on which two illustrated objects could be expected as potential sentence continuations. The gaze cue only additionally directed attention to one of the two objects, but importantly, at that point both potential targets were already activated. Thus, when one of the two targets was mentioned, the comprehension followed smoothly. No disruption occurred when the object that was not cued was mentioned. In contrast, further activating an object with a gaze cue, and finally mentioning it, showed to be facilitatory for the comprehension of the linguistic reference.

Staudte and Crocker (2011) found both an effect of congruent gaze facilitation, and a disruption caused by the incongruent gaze cue. Their study made use of sentences with comparatives which inspired anticipation of a certain object (*The cylinder is taller than the pyramid that is pink*.). Incongruent gaze cue was directed to an object of the same type, but of different size (tall brown pyramid), rendering the gaze cue not only incongruent, but also mismatched with the previous linguistic context. We, on the other hand, used a linguistic context that activated two of the presented four objects, rendering them equally plausible to be mentioned next. Consequently, a gaze cue to one of those objects, even when incongruent with the upcoming noun, was not disruptive, since it was plausible given the context, highlighting an object already activated by the verbal constraint.

We conclude that the different results found in our experiment reflect the interplay of the linguistic and visual cues that are not present in the mentioned studies. On the one hand, when the gaze cue is not only incongruent with the subsequent target, but also mismatched with the previous linguistic context (as in Staudte & Crocker, 2011), it is not clear which of the two factors are driving the effect. On the other hand, when referential gaze cue is the only piece of information that inspires an anticipation for the target object, its incongruency turns out to be costly (Jachmann et al., 2017; Macdonald & Tatler, 2014). However, as we have shown, when the gaze cue is there as an additional cue to language, the linguistic information is not discarded once gaze is introduced; rather, they are considered together until the reference is resolved.

## 5 Results summary and conclusions

Three studies were conducted in order to gain more insight into the immediate effect of referential gaze cue on language processing. We examined listeners’ visual attention during simultaneous presentation of both linguistic and visual stimuli. In addition, we measured immediate cognitive effort required to perceive and process the information coming from both modalities, in absence of a behavioral task. We summarize the findings of the presented studies by referring back to the questions raised at the very beginning of the paper. Moreover, Table 7 gives a short overview of the main gaze cue effects measured on the cognitive load.

**Table 7:** Summary of the main cognitive load results

		Gaze window	Reference window
**Experiment 1:**			
*no gaze* vs. *congruent gaze*		n.s.	no gaze > congruent gaze
**Experiment 2:**		**Part 1**	
*no gaze*	*verb fitting*	gaze × fit	n.s.
vs.	vs.	**Part 2**	
*congruent gaze*	*mismatched*	no gaze > congruent gaze	no gaze > congruent gaze
**Experiment 3:**			
*no gaze* vs. *congruent gaze* vs. *incongruent gaze*		n.s.	no gaze > congruent gaze no gaze = incongruent gaze

First, we found evidence that the gaze cue influences the predictability of linguistic reference. All three conducted experiments indicate that gaze is followed; that is, it leads to a shift in visual attention, regardless of its fit to the linguistic context or its slightly reduced reliability. This subsequently influences cognitive effort required for processing the reference.

Second, we found that the existence of the gaze cue facilitates processing of the subsequent referent noun. Experiment 1 manipulated the existence of the (fitting and congruent) gaze cue and found that it is always followed, reducing the load on the reference, while maintaining the facilitation for the most plausible referent. Experiment 2 went a step further and examined a congruent, but mismatching cue. Again, gaze led to a (slight) reduction of cognitive load on the referent noun, even when both the cue and the noun mismatched the linguistic context. Importantly, though, this effect was found only upon establishing trust in the gaze cue, that is, adapting to the sometimes anomalous gaze. Experiment 3 manipulated gaze congruency and found, again, a benefit of congruent gaze cues, but, interestingly, no cost of incongruent cues. In addition, eye movement analysis has shown that even though gaze was followed and made one object more salient, the other plausible object was not dropped from attention until the reference was uttered.

Third, we wondered about the immediate cost of gaze cue perception and utilization. Experiments 1 and 3 give evidence that a gaze cue fitting the linguistic context does not induce higher cognitive effort. However, Experiment 2, which examined gaze cuing an object that does not fit the previous context, has shown evidence that such anomalous gaze indeed induces immediate cost. This suggests that the cost is induced when the visual cue cannot be incorporated with the previously established linguistic context.

Fourth, inspired by the UID hypothesis, we had expected to detect a distribution of cognitive effort between the linguistic and visual cues. All three experiments showed that the existence of a fitting congruent gaze cue leads to the reduction of cognitive effort on the reference. However, this reduction was not preceded by an increase in cognitive effort induced by perceiving the gaze cue itself. Interestingly, though, Experiment 2 indeed showed some evidence for such a distribution, where the surprising gaze cue induced higher cognitive load, which was followed by the reduction of effort on the referent noun (second half of the experiment). The same condition without the gaze cue showed that the referent noun bore all the effort required for processing the anomalous sentence.

Finally, our findings suggest that the gaze cue is strategically used. It further highlights an object but does not inhibit previous activation of a different object. Thus, gaze is not disruptive when incongruent with the linguistic reference (given that the reference fits the context).

The question arises as to whether the gaze cue used in the present experiments is actually more similar to a visual pointer, like an arrow cue, than an actual human gaze cue. A body of research has addressed the difference between gaze and other visual pointers like arrows. Friesen, Ristic, and Kingstone (2004) found that eyes trigger an initial reflexive attention shift to the cued location, which is not suppressed in contexts where the opposite direction is the relevant one. In contrast, they found that such a reflexive attention shift was avoidable with arrow cues. The authors conclude that the attention effect for eyes is more strongly reflexive than that for arrows (Ristic, Wright, & Kingstone, 2007). Staudte et al. (2014) initially found evidence that reverse gaze cuing disrupted comprehension, while reverse arrows facilitated it. However, once gaze was made as precise as arrows, no qualitative difference was found in their influence on comprehension (Staudte et al., 2014). The authors conclude that a beneficial effect of object‐oriented speaker gaze can potentially be replicated by any other visual cue. In addition, Böckler, Knoblich, and Sebanz (2011) examine gaze‐following and shared attention, and find that shared attention occured both for schematic and for real faces (photographs of humans), also not depending on the spatial arrangement of the two observed faces, or their spatial arrangement in relation to the participant. Hence, we consider our gaze cue as an abstract and strategic visual cue, not being concerned with the difference between an actual gaze and a different visual pointer, but understanding that our findings reflect the state of affairs for visual cues in general.

In sum, our assessment of anticipatory eye movements, visual attention shifts and immediate cognitive load allowed for gaining novel insight into the interplay between visual perception and language processing. Our findings are in line with previous research showing that verb selectional preferences inspire activation of context‐fitting visually presented objects, while gaze cue can further highlight the same, or lead to a shift in visual attention to an alternative object—such a shift happens even when the gaze is cuing an object not fitting the context. Also, as was previously assessed employing behavioral tasks, we found that the referential gaze cue indeed facilitates reference processing. Importantly, however, we showed this by measuring cognitive effort online, as it is induced, and without having to rely on a secondary task. Our findings suggest that the gaze cue reduces cognitive load required for processing the corresponding linguistic reference even when they are both not fitting the previous context. In addition, we were able to further extend the current understanding of gaze cue perception by showing that the facilitatory effect of gaze on language processing is not preceded by an additional cost of perceiving the gaze cue. The perception of the gaze cue does not induce higher cognitive effort when it fits the context (i.e., when objects of different probability are cued); only mismatching, anomalous gaze showed to be costly. Interestingly, we observed that the gaze cue always led to a shift in visual attention, which was, however, not always coupled with the same cognitive effort. This finding further suggests that visual attention is not indicative of the intensity of the induced processing effort.

## Supporting information


**Table S1.** Exp. 1—Linguistic stimuli. Constraint was manipulated by verb restrictiveness, and Plausibility by noun fit with the restrictive verb.

**Table S2.** Exp. 1—Visual stimuli given in the state prior to the gaze cue.

**Table S3.** Exp. 2—Linguistic stimuli (version A). Fit was manipulated by whether the referent noun fits the verb.

**Table S4.** Exp. 2—Linguistic stimuli (version B). Fit was manipulated by whether the referent noun fits the verb.

**Table S5.** Exp. 2—Visual stimuli given in the state prior to the gaze cue.

**Table S6.** Exp. 3—Linguistic stimuli (versions A and B).

**Table S7.** Exp. 3—Visual stimuli given in the state prior to the gaze cue.

**Fig. S1.** Exp. 1—New inspections of *water*,* ice cream*, and two distractor objects (95% CI error bars).
**Fig. S2.** Exp. 2—New inspections of *water*,* sausage*, and distractors when *sausage* or *water* are gazed at (right) versus no‐gaze condition (95% CI error bars).
**Fig. S3.** Exp. 2—Proportion of fixations in the two halves of the experiment.
**Fig. S4.** Exp. 2—New inspections of target and competitor, in the gaze region of interest (95% CI error bars).
**Fig. S5.** Exp. 3—New inspections of the four presented objects in the gaze region of interest (95% CI error bars).

## Notes

1 Throughout the text this potential effect is referred to as the load induced by *the gaze cue itself*. However, note that we do not mean the load of perceiving the face, or the movement of the eyes, but rather, the load induced by considering the object cued by the gaze.

2 RatingScore ∼ Const × Plaus + (1 + Const + Plaus ‖ Subject) + (1 + Const × Plaus ‖ Item), family = Poisson (link = “log”)

3 RatingScore ∼ Plaus + (1 + Plaus | Subject) + (1 + Plaus | Item), family = Poisson (link = “log”)

4 An exhaustive list of all 20 item sentences presented in four conditions each is given in Table S1 in Supporting Information.

5 The list of visual displays used for each item is given in Table S2 in Supporting Information.

6 This is the required setup for the extraction of the ICA events from EyeWorks Workload Module software (version 3.12).

7 Average noun duration in Exp. 1: 596 ms (*SD* = 106.05), Exp. 2: 620 ms (*SD* = 118.13), Exp. 3: 632 ms (*SD* = 109.73).

8 The results are illustrated by Fig. 1 given in Figures S1–S5 in Supporting Information.

9 The independent variable Half is first relevant for the analysis of Experiment 2. Hence, it will be justified in the following section (see Section 3). Here, as well as in Experiment 3, Half is included in the analyses for the sake of consistency among the experiments.

10 All 20 items are given in Table S3 (version A) and Table S4 (version B) in Supporting Information.

11 All visual displays used for this experiment are given in Table S5 in Supporting Information.

12 This was done for both versions of the experiment.

13 The results are illustrated by Fig. 2 given in Figures S1–S5 in Supporting Information.

14 Illustrated by Fig. 3 given in Figures S1–S5 in Supporting Information.

15 Fig. 4 given in Figures S1–S5 in Supporting Information shows that while new inspections of *water* pattern similarly in the two parts, new inspections to *sausage* are slightly reduced in the second half of the experiment. Thus, even though the gaze cue constantly led to a shift in visual attention, in the second half, the inspection of the fitting object *water* was not as inhibited after the gaze to *sausage* as was the case in the first part. That is, in the second half, upon a gaze cue to *sausage* a new inspection was equally likely to be directed to either *sausage* or *water*.

16 The list of all items is given in Table S6 (both versions) in Supporting Information.

17 All visual displays used for this experiment are given in Table S7 in Supporting Information.

18 The results are illustrated in Fig. 5 given in Figures S1–S5 in Supporting Information.

## Appendix A Further Comparisons

**Table A1:** Exp. 1—Further comparisons for new inspections (gaze region of interest)

	(a) subset *water*				(b) subset *ice cream*			
Predictor	β	*SE*	*z*	*p*	β	*SE*	*z*	*p*
1. Target inspections								
Intercept	−1.597	0.089	−17.959	<2e−16***	−2.032	0.092	−22.135	<2e−16***
Gaze	0.648	0.178	3.641	0.0003***	−0.150	0.184	−0.815	0.415
2. Competitor inspections								
Intercept	−2.788	0.175	−15.957	<2e−16***	−1.752	0.098	−17.877	<2e−16***
Gaze	−0.204	0.296	−0.688	0.491	0.721	0.187	3.853	0.0001***

*Notes*: ****p* < .001.

(a,b) Target ∼ Gaze + (1 + Gaze | Subject) + (1 | Item), family = “binomial”).

(c) Competitor ∼ Gaze + (1 + Gaze ‖ Subject) + (1 | Item), family = “binomial”).

(d) Competitor ∼ Gaze + (1 + Gaze | Subject) + (1 | Item), family = “binomial”).

**Table A2:** Exp. 1—Further comparisons for the ICA in the subsets of the two verbs

	(a) subset *spill*				(b) subset *order*			
Predictor	β	*SE*	*z*	*p*	β	*SE*	*z*	*p*
1. Gaze window								
Intercept	2.877	0.030	96.67	<2e−16***	2.880	0.0314	91.80	<2e−16***
Plausibility	0.051	0.031	1.62	0.104	−0.013	0.027	−0.49	0.624
Gaze	−0.003	0.055	−0.05	0.957	0.012	0.060	0.20	0.842
Half	−0.018	0.019	−0.96	0.338	−0.044	0.019	−2.30	0.022*
2. Reference window								
Intercept	2.897	0.030	97.03	<2e−16***	2.937	0.028	103.54	<2e−16***
Plausibility	0.152	0.035	4.37	1.23e−05***	−0.043	0.037	−1.15	0.249
Gaze	−0.148	0.056	−2.63	0.009**	−0.103	0.054	−1.93	0.054 .
Half	−0.049	0.019	−2.59	0.010**	−0.044	0.019	−2.39	0.017*

*Notes*: .*p* < .1,**p* < .05, ***p* < .01, ****p* < .001.

(a,b) ICA ∼ Plausibility + Gaze + Half + (1 + Plausibility | Subject) + (1 + Plausibility | Item), family = Poisson (link = “log”).

**Table A3:** Exp. 2—Further comparisons for new inspections of *sausage*

	(a) subset no‐gaze				(b) subset ref. gaze			
Predictor	β	*SE*	*z*	*p*	β	*SE*	*z*	*p*
*sausage* inspections								
Intercept	−2.699	0.201	−13.445	<2e−16***	−2.186	0.168	−12.993	<2e−16***
Fit	0.077	0.351	0.220	0.826	1.288	0.279	4.618	3.87e−06***
Half	−0.645	0.353	−1.825	0.068 .	−0.312	0.254	−1.227	0.22

*Notes*: .*p* < .1, ****p* < .001.

(a) Competitor ∼ Fit + Half (1 + Fit + Half ‖ Subject) + ( 1 + Fit | Item), family = “binomial”).

(b) Competitor ∼ Fit + Half (1 + Fit + Half ‖ Subject) + ( 1 + Fit ‖ Item), family = “binomial”).

**Table A4:** Exp. 2—Further comparisons for the ICA in the subsets of the two halves of the experiment for both the gaze time window and the reference time window

	(a) subset 1st half				(b) subset 2nd half			
Predictor	β	*SE*	*z*	*p*	β	*SE*	*z*	*p*
1. Gaze time window								
Intercept	2.783	0.044	63.28	<2e−16***	2.751	0.047	58.76	<2e−16***
Gaze	0.020	0.048	0.43	0.6670	0.099	0.045	2.20	0.028*
Fit	0.051	0.066	0.77	0.440	0.013	0.048	0.27	0.784
Gaze:Fit	0.180	0.084	2.13	0.033*	−0.008	0.090	−0.09	0.929
2. Reference time window								
Intercept	2.803	0.039	71.72	<2e−16***	2.745	0.044	61.95	<2e−16***
Gaze	0.043	0.051	0.86	0.392	−0.091	0.047	−1.93	0.054 .
Fit	0.199	0.064	3.11	0.002**	0.250	0.067	3.76	0.0002***
Gaze:Fit	−0.059	0.092	−0.64	0.520	−0.026	0.073	−0.35	0.726

*Notes*: .*p* < .1,**p* < .05, ***p* < .01, ****p* < .001.

1(a,b) ICA ∼ Gaze × Fit + (1 + Gaze × Fit ‖ Subject) + (1 + Fit | Item), family = Poisson (link = “log”).

2(a,b) ICA ∼ Gaze × Fit + (1 + Gaze × Fit | Subject) + (1 + Fit | Item), family = Poisson (link = “log”).
