# Supplementary material for: Eye’ll Help You Out! How the Gaze Cue Reduces the Cognitive Load Required for Reference Processing
Source: Cogn Sci. 2018 Oct 7;42(8):2418–58. doi: 10.1111/cogs.12682 (PMC6585668; doi:10.1111/cogs.12682)
Supplement: Supplementary file 1 — Table S1. Exp. 1—Linguistic stimuli. Constraint was manipulated by verb restrictiveness, and Plausibility by noun fit with the restrictive verb. [file COGS-42-2418-s001.pdf]

Exp. 1 – Linguistic Stimuli. Constraint was manipulated by verb restrictiveness, and Plausibility by noun fit with the restrictive verb.

Item	Verb	Object	Sentence
1	1	1	Die Mutter löffelt gleich die Suppe.
1	1	2	Die Mutter löffelt gleich den Kaffee.
1	2	1	Die Mutter bewertet gleich die Suppe.
1	2	2	Die Mutter bewertet gleich den Kaffee.
2	1	1	Der Großvater verschüttet gleich das Wasser.
2	1	2	Der Großvater verschüttet gleich das Eis.
2	2	1	Der Großvater bestellt gleich das Wasser.
2	2	2	Der Großvater bestellt gleich das Eis.
3	1	1	Die Schwester schmilzt gleich die Butter.
3	1	2	Die Schwester schmilzt gleich den Honig.
3	2	1	Die Schwester kontrolliert gleich die Butter.
3	2	2	Die Schwester kontrolliert gleich den Honig.
4	1	1	Die Cousine montiert gleich die Antenne.
4	1	2	Die Cousine montiert gleich das Motorrad.
4	2	1	Die Cousine ersetzt gleich die Antenne.
4	2	2	Die Cousine ersetzt gleich das Motorrad.
5	1	1	Der Großvater kocht gleich die Kartoffel.
5	1	2	Der Großvater kocht gleich die Banane.
5	2	1	Der Großvater verpackt gleich die Kartoffel.
5	2	2	Der Großvater verpackt gleich die Banane.
6	1	1	Der Großmutter trinkt gleich den Kaffee.
6	1	2	Der Großmutter trinkt gleich den Joghurt.
6	2	1	Der Großmutter testet gleich den Kaffee.
6	2	2	Der Großmutter testet gleich den Joghurt.
7	1	1	Der Großvater serviert gleich das Eis.
7	1	2	Der Großvater serviert gleich die Zitrone.
7	2	1	Der Großvater fotografiert gleich das Eis.
7	2	2	Der Großvater fotografiert gleich die Zitrone.
8	1	1	Der Großvater isst gleich das Brot.
8	1	2	Der Großvater isst gleich den Pfeffer.
8	2	1	Der Großvater nimmt gleich das Brot.
8	2	2	Der Großvater nimmt gleich den Pfeffer.
9	1	1	Der Cousin poliert gleich das Auto.
9	1	2	Der Cousin poliert gleich den Zug.
9	2	1	Der Cousin erblickt gleich das Auto.
9	2	2	Der Cousin erblickt gleich den Zug.
10	1	1	Der Vater kühlt gleich den Wein.
10	1	2	Der Vater kühlt gleich die Waffel.
10	2	1	Der Vater reklamiert gleich den Wein.
10	2	2	Der Vater reklamiert gleich die Waffel.
11	1	1	Die Schwester bestickt gleich das Kissen.
11	1	2	Die Schwester bestickt gleich den Stiefel.
11	2	1	Die Schwester behalt gleich das Kissen.
11	2	2	Die Schwester behalt gleich den Stiefel.
12	1	1	Der Großmutter zuckert gleich den Tee.
12	1	2	Der Großmutter zuckert gleich die Zitrone.
12	2	1	Der Großmutter pruft gleich den Tee.
12	2	2	Der Großmutter pruft gleich die Zitrone.
13	1	1	Der Mann fährt gleich das Auto.
13	1	2	Der Mann fährt gleich das Schiff.
13	2	1	Der Mann sieht gleich das Auto.
13	2	2	Der Mann sieht gleich das Schiff.
14	1	1	Der Cousin näht gleich die Jacke.
14	1	2	Der Cousin näht gleich das Sofa.
14	2	1	Der Cousin berührt gleich die Jacke.
14	2	2	Der Cousin berührt gleich das Sofa.
15	1	1	Die Frau schneidet gleich das Brot.
15	1	2	Die Frau schneidet gleich die Pommes.
15	2	1	Die Frau holt gleich das Brot.
15	2	2	Die Frau holt gleich die Pommes.
16	1	1	Die Mutter flickt gleich die Jeans.
16	1	2	Die Mutter flickt gleich den Besen.
16	2	1	Die Mutter vergisst gleich die Jeans.
16	2	2	Die Mutter vergisst gleich den Besen.
17	1	1	Die Frau bügelt gleich das T-Shirt.
17	1	2	Die Frau bügelt gleich die Socke.
17	2	1	Die Frau beschreibt gleich das T-Shirt.
17	2	2	Die Frau beschreibt gleich die Socke.
18	1	1	Die Mutter strickt gleich den Schal.
18	1	2	Die Mutter strickt gleich die Decke.
18	2	1	Die Mutter bekommt gleich den Schal.
18	2	2	Die Mutter bekommt gleich die Decke.
19	1	1	Die Frau erntet gleich den Apfel.
19	1	2	Die Frau erntet gleich den Reis.
19	2	1	Die Frau wäscht gleich den Apfel.
19	2	2	Die Frau wäscht gleich den Reis.
20	1	1	Der Bruder repariert gleich den Laptop.
20	1	2	Der Bruder repariert gleich das Raumschiff.
20	2	1	Der Bruder zeichnet gleich den Laptop.
20	2	2	Der Bruder zeichnet gleich das Raumschiff.
