# Supplementary material for: Eye’ll Help You Out! How the Gaze Cue Reduces the Cognitive Load Required for Reference Processing
Source: Cogn Sci. 2018 Oct 7;42(8):2418–58. doi: 10.1111/cogs.12682 (PMC6585668; doi:10.1111/cogs.12682)

Exp. 1 – Visual stimuli given in the state prior to the gaze cue.

Item 1

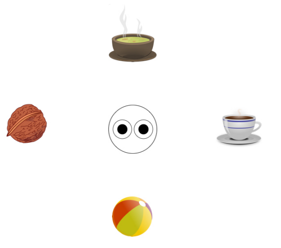

Item 2

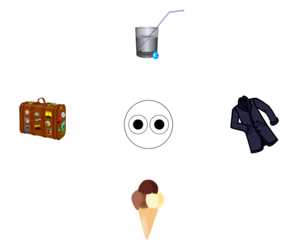

Item 3

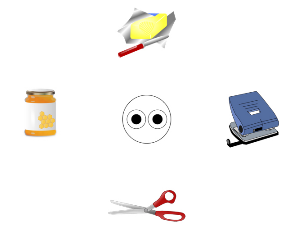

Item 4

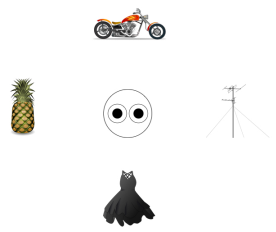

Item 5

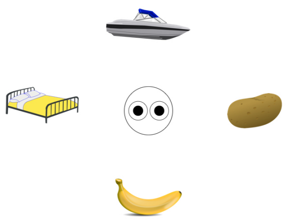

Item 6

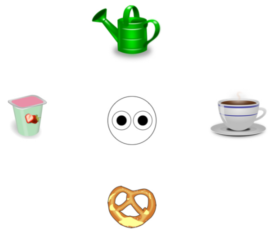

Item 7

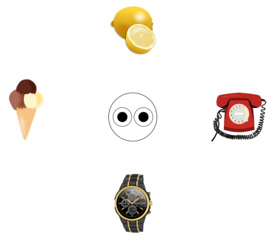

Item 8

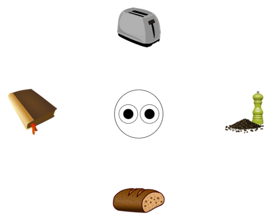

Item 9

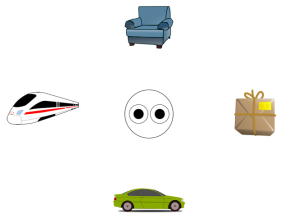

Item 10

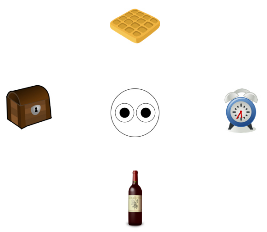

Item 11

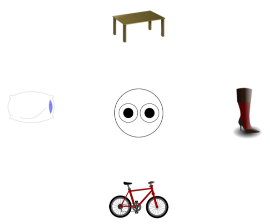

Item 12

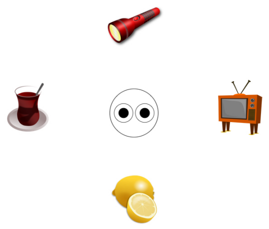

[0]Clip arts have been retrieved from www.openclipart.org or www.pixabay.com

Item 13

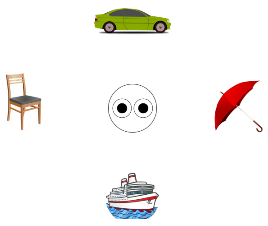

Item 14

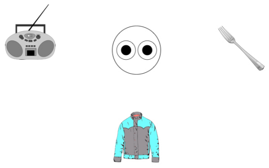

Item 15

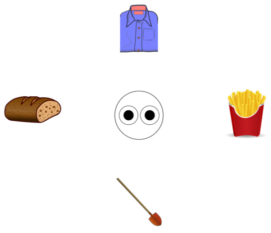

Item 16

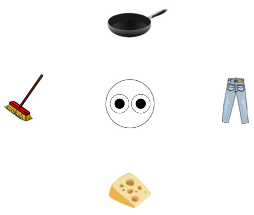

Item 17

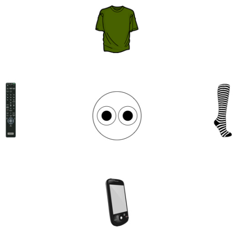

Item 18

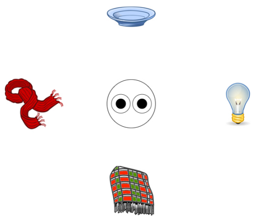

Item 19

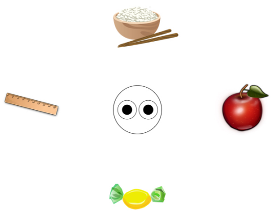

Item 20

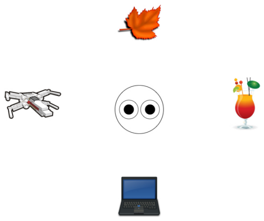

Supplement: Supplementary file 2 — Table S2. Exp. 1—Visual stimuli given in the state prior to the gaze cue. [file COGS-42-2418-s002.pdf]
