# Supplementary material for: Eye’ll Help You Out! How the Gaze Cue Reduces the Cognitive Load Required for Reference Processing
Source: Cogn Sci. 2018 Oct 7;42(8):2418–58. doi: 10.1111/cogs.12682 (PMC6585668; doi:10.1111/cogs.12682)
Supplement: Supplementary file 3 — Table S3. Exp. 2—Linguistic stimuli (version A). Fit was manipulated by whether the referent noun fits the verb. [file COGS-42-2418-s003.pdf]

Exp. 2 – Linguistic Stimuli (version A). Fit was manipulated by whether the referent noun fits the verb.

Item	Object	Sentence
1	1	Der Mann löffelt gleich die Suppe.
1	2	Der Mann löffelt gleich den Schuh.
2	1	Der Mann verschüttet gleich das Wasser.
2	2	Der Mann verschüttet gleich die Wurst.
3	1	Der Mann schmilzt gleich die Butter.
3	2	Der Mann schmilzt gleich den Schrank.
4	1	Der Mann montiert gleich die Antenne.
4	2	Der Mann montiert gleich die Rose.
5	1	Die Frau kocht gleich die Kartoffel.
5	2	Die Frau kocht gleich den Stuhl.
6	1	Die Frau trinkt gleich den Kaffee.
6	2	Die Frau trinkt gleich die Zwiebel.
7	1	Die Frau serviert gleich das Eis.
7	2	Die Frau serviert gleich die Büroklammer.
8	1	Der Mann isst gleich die Waffel.
8	2	Der Mann isst gleich die Zeitung.
9	1	Der Mann poliert gleich das Auto.
9	2	Der Mann poliert gleich den Salat.
10	1	Die Frau kühlt gleich den Wein.
10	2	Die Frau kühlt gleich den Buntstift.
11	1	Die Frau bestickt gleich das Kissen.
11	2	Die Frau besticktt gleich den Topf.
12	1	Der Mann zuckert gleich den Tee.
12	2	Der Mann zuckert gleich den Hubschrauber.
13	1	Die Frau fährt gleich das Auto.
13	2	Die Frau fährt gleich das Klavier.
14	1	Die Frau näht gleich die Jacke.
14	2	Die Frau näht gleich die Nuss.
15	1	Der Mann filetiert gleich den Fisch.
15	2	Der Mann filetiert gleich die Geige.
16	1	Die Frau flickt gleich die Jeans.
16	2	Die Frau flickt gleich die Blume.
17	1	Die Frau bügelt gleich das T-Shirt.
17	2	Die Frau bügelt gleich den Keks.
18	1	Die Frau strickt gleich den Schal.
18	2	Die Frau strickt gleich die Orange.
19	1	Der Mann mauert gleich die Wand.
19	2	Der Mann mauert gleich den Koffer.
20	1	Der Mann repariert gleich den Laptop.
20	2	Der Mann repariert gleich die Zigarre.
