# Supplementary material for: Eye’ll Help You Out! How the Gaze Cue Reduces the Cognitive Load Required for Reference Processing
Source: Cogn Sci. 2018 Oct 7;42(8):2418–58. doi: 10.1111/cogs.12682 (PMC6585668; doi:10.1111/cogs.12682)
Supplement: Supplementary file 4 — Table S4. Exp. 2—Linguistic stimuli (version B). Fit was manipulated by whether the referent noun fits the verb. [file COGS-42-2418-s004.pdf]

Exp. 2 – Linguistic Stimuli (version B). Fit was manipulated by whether the referent noun fits the verb.

Item	Object	Sentence
1	1	Der Mann bindet gleich den Schuh.
1	2	Der Mann bindet gleich die Suppe.
2	1	Der Mann grillt gleich die Wurst.
2	2	Der Mann grillt gleich das Wasser.
3	1	Der Mann verleimt gleich den Schrank.
3	2	Der Mann verleimt gleich die Butter.
4	1	Der Mann pflanzt gleich die Rose.
4	2	Der Mann pflanzt gleich die Antenne.
5	1	Die Frau streicht gleich den Stuhl.
5	2	Die Frau streicht gleich die Kartoffel.
6	1	Die Frau schält gleich die Zwiebel.
6	2	Die Frau schält gleich den Kaffee.
7	1	Die Frau verbiegt gleich die Büroklammer.
7	2	Die Frau verbiegt gleich das Eis.
8	1	Der Mann ließt gleich die Zeitung.
8	2	Der Mann ließt gleich die Waffel.
9	1	Der Mann würzt gleich den Salat.
9	2	Der Mann würzt gleich das Auto.
10	1	Die Frau spitzt gleich den Buntstift.
10	2	Die Frau spitzt gleich den Wein.
11	1	Die Frau spült gleich den Topf.
11	2	Die Frau spült gleich das Kissen.
12	1	Der Mann lenkt gleich den Hubschrauber.
12	2	Der Mann lenkt gleich den Tee.
13	1	Die Frau stimmt gleich das Klavier.
13	2	Die Frau stimmtgleich das Auto.
14	1	Die Frau knackt gleich die Nuss.
14	2	Die Frau knackt gleich die Jacke.
15	1	Der Mann spielt gleich die Geige.
15	2	Der Mann spielt gleich den Fisch.
16	1	Die Frau gießt gleich die Blume.
16	2	Die Frau gießt gleich die Jeans.
17	1	Die Frau bäckt gleich den Keks.
17	2	Die Frau bäckt gleich das T-Shirt.
18	1	Die Frau entsaftet gleich die Orange.
18	2	Die Frau entsaftet gleich den Schal.
19	1	Der Mann packt gleich den Koffer.
19	2	Der Mann packt gleich die Wand.
20	1	Der Mann raucht gleich die Zigarre.
20	2	Der Mann raucht gleich den Laptop.
