# Supplementary material for: Eye’ll Help You Out! How the Gaze Cue Reduces the Cognitive Load Required for Reference Processing
Source: Cogn Sci. 2018 Oct 7;42(8):2418–58. doi: 10.1111/cogs.12682 (PMC6585668; doi:10.1111/cogs.12682)
Supplement: Supplementary file 6 — Table S6. Exp. 3—Linguistic stimuli (versions A and B). [file COGS-42-2418-s006.pdf]

Exp. 3 – Linguistic Stimuli (versions A and B).

Item	Version	Sentence
1	A	Die Frau dünstet gleich den Pilz.
1	B	Die Frau dünstet gleich die Paprika.
2	A	Der Mann sät gleich den Weizen.
2	B	Der Mann sät gleich den Mais.
3	A	Der Mann versichert gleich das Handy.
3	B	Der Mann versichert gleich das Fahrrad.
4	A	Die Frau etikettiert gleich die Marmelade.
4	B	Die Frau etikettiert gleich die Flashe.
5	A	Die Frau trocknet gleich die Gabel.
5	B	Die Frau trocknet gleich den Löffel.
6	A	Die Frau frühstückt gleich den Pfannkuchen.
6	B	Die Frau frühstückt gleich das Obst.
7	A	Der Mann nascht gleich das Gummibärchen.
7	B	Der Mann nascht gleich das Bonbon.
8	A	Der Mann schneidet gleich den Kuchen.
8	B	Der Mann schneidet gleich die Pizza.
9	A	Die Frau grillt gleich die Wurst.
9	B	Die Frau grillt gleich das Steak.
10	A	Die Frau kocht gleich die Zucchini.
10	B	Die Frau kocht gleich die Aubergine.
11	A	Die Frau bäckt gleich den Keks.
11	B	Die Frau bäckt gleich den Muffin.
12	A	Der Mann spielt gleich das Akkordeon.
12	B	Der Mann spielt gleich das Saxofon.
13	A	Die Frau spült gleich den Topf.
13	B	Die Frau spült gleich den Teller.
14	A	Die Frau schält gleich die Kartoffel.
14	B	Die Frau schält gleich die Zwiebel.
15	A	Der Mann bindet gleich den Schuh.
15	B	Der Mann bindet gleich die Krawatte.
16	A	Der Mann würzt gleich den Salat.
16	B	Der Mann würzt gleich die Suppe.
17	A	Der Mann isst gleich die Waffel.
17	B	Der Mann isst gleich das Croissant.
18	A	Der Mann kühlt gleich den Wein.
18	B	Der Mann kühlt gleich das Bier.
19	A	Die Frau serviert gleich das Eis.
19	B	Die Frau serviert gleich die Torte.
20	A	Der Mann verschüttet gleich den Tee.
20	B	Der Mann verschüttet gleich den Saft.
21	A	Der Mann zuckert gleich den Tee.
21	B	Der Mann zuckert gleich den Kaffee.
