# Supplementary material for: Eye’ll Help You Out! How the Gaze Cue Reduces the Cognitive Load Required for Reference Processing
Source: Cogn Sci. 2018 Oct 7;42(8):2418–58. doi: 10.1111/cogs.12682 (PMC6585668; doi:10.1111/cogs.12682)
Supplement: Supplementary file 8 — Fig. S1. Exp. 1—New inspections of water, ice cream, and two distractor objects (95% CI error bars). Fig. S2. Exp. 2—New inspections of water, sausage, and distractors when sausage or water are gazed at (right) versus no‐gaze condition (95% CI error bars). Fig. S3. Exp. 2—Proportion of fixations in the two halves of the experiment. Fig. S4. Exp. 2—New inspections of target and competitor, in the gaze region of interest (95% CI error bars). Fig. S5. Exp. 3—New inspections of the four presented objects in the gaze region of interest (95% CI error bars). [file COGS-42-2418-s008.pdf]

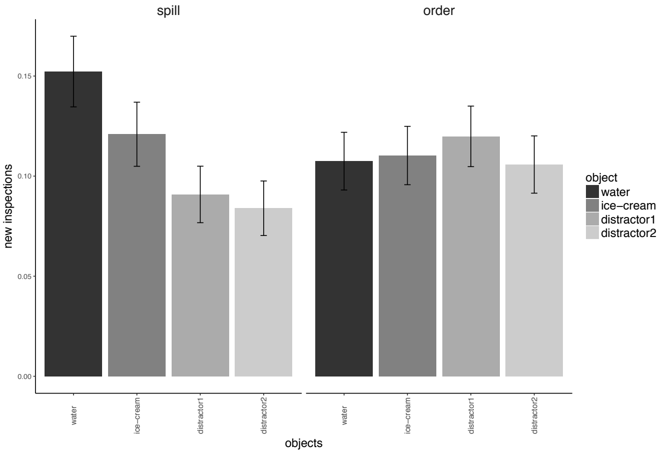

(a) Verb region of interest: *spill* (left) and *order* (right).

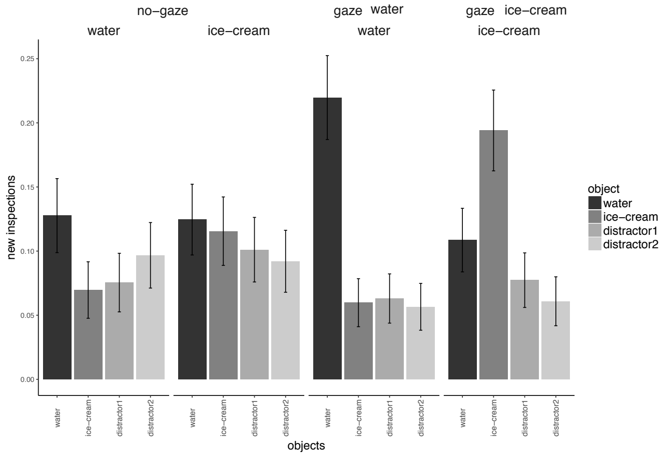

(b) Gaze region of interest: gaze cuing *water* or *ice cream*: no-gaze (left) and referent gaze (right) condition.

Fig. 1. Exp. 1 – New inspections of *water*, *ice cream* and two distractor objects (95% CI error bars).

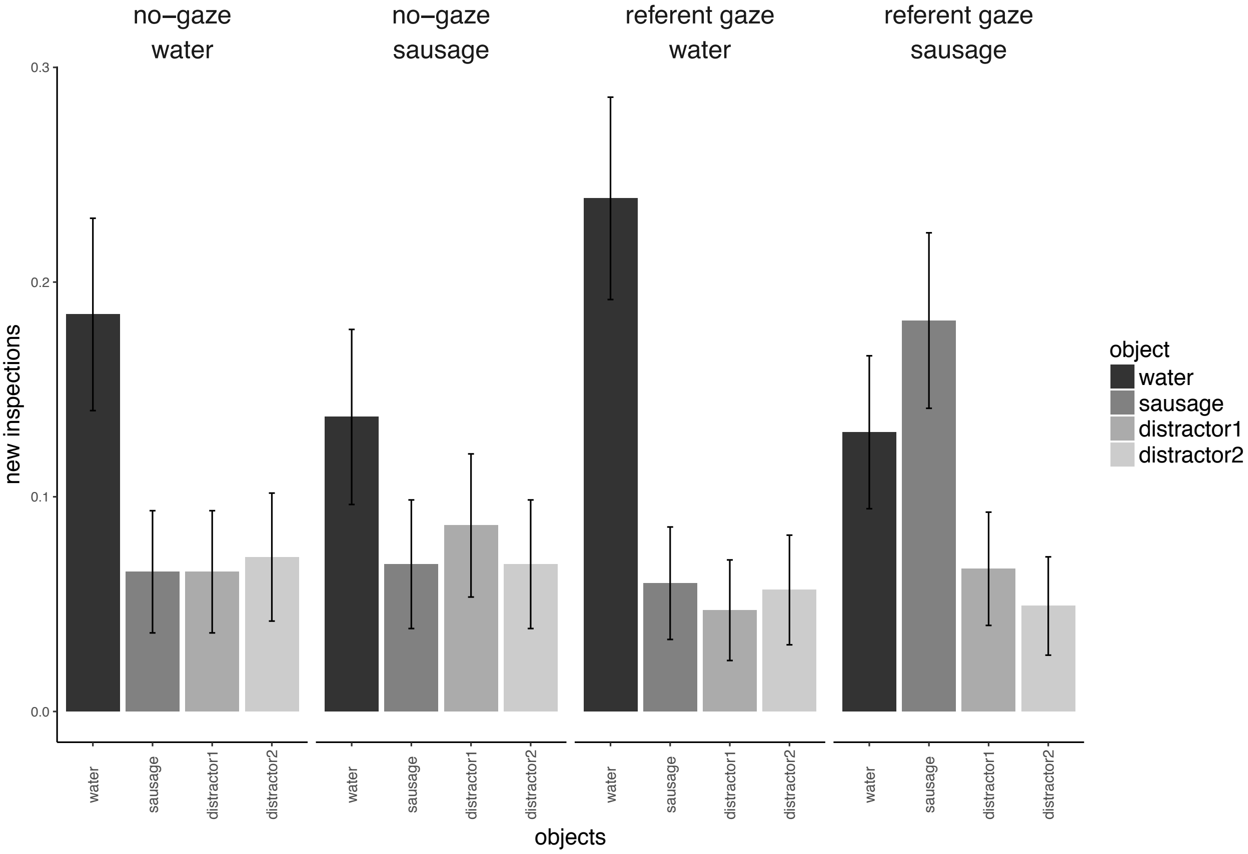

Fig. 2. Exp. 2 – New inspections of *water*, *sausage* and distractors when *sausage* or *water* are gazed at (right) vs. no-gaze condition (95% CI error bars).

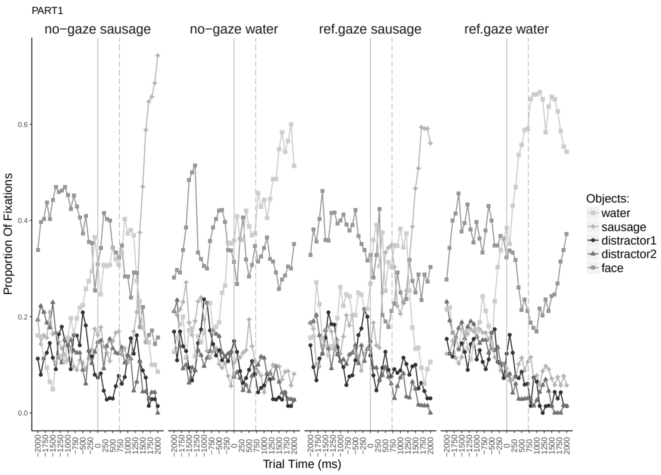

(a) 1st half of the experiment

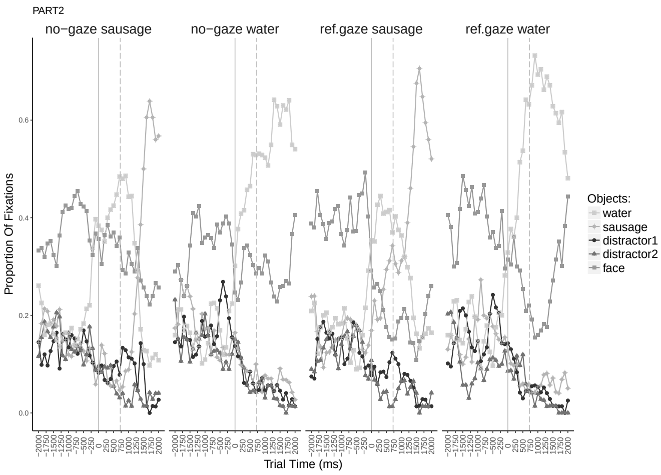

(b) 2nd half of the experiment

Fig. 3. Exp. 2 – Proportion of Fixations in the two halves of the experiment.

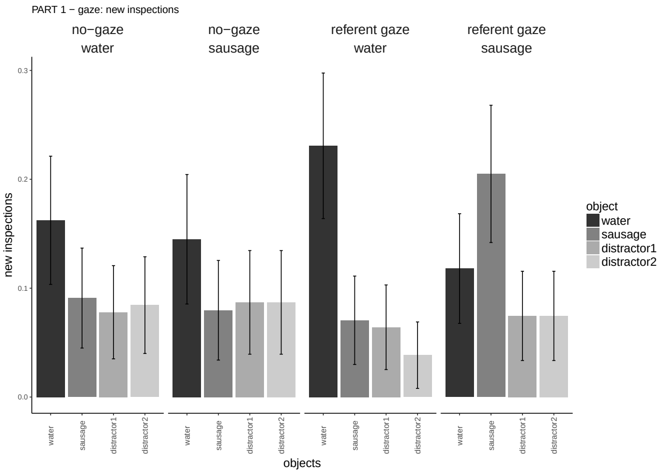

(a) 1st half of the experiment

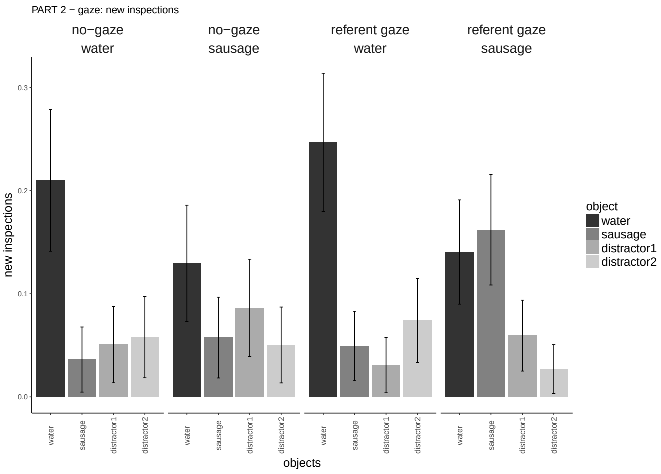

(b) 2nd half of the experiment

Fig. 4. Exp. 2 – New inspections of target and competitor, in the gaze region of interest (95% CI error bars).

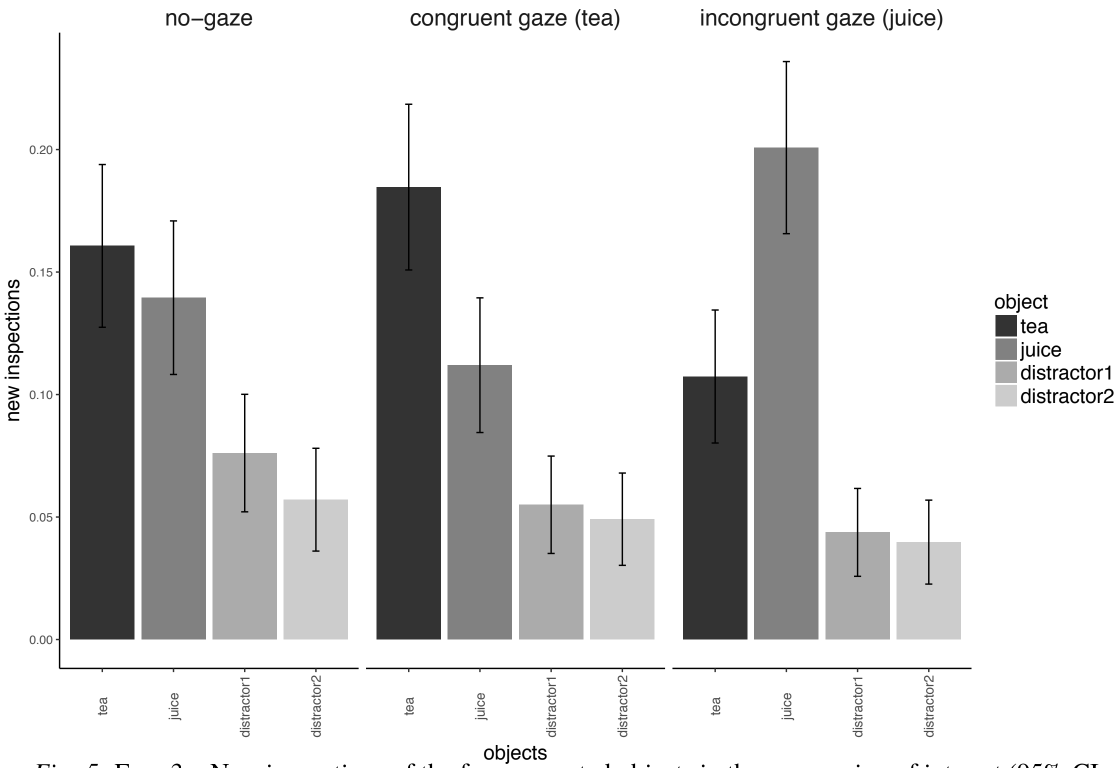

Fig. 5. Exp. 3 – New inspections of the four presented objects in the gaze region of interest (95% CI error bars).
